# Microbial dynamics with CRC progression: a study of the mucosal microbiota at multiple sites in cancers, adenomatous polyps, and healthy controls

**DOI:** 10.1007/s10096-023-04551-7

**Published:** 2023-01-27

**Authors:** Thulasika Senthakumaran, Aina E. F. Moen, Tone M. Tannæs, Alexander Endres, Stephan A. Brackmann, Trine B. Rounge, Vahid Bemanian, Hege S. Tunsjø

**Affiliations:** 1 Department of Life Sciences and Health, Oslo Metropolitan University, Oslo, Norway; 2 Section for Clinical Molecular Biology (EpiGen), Akershus University Hospital, Lørenskog, Norway; 3 Department of Clinical Molecular Biology, Institute of Clinical Medicine, University of Oslo, Oslo, Norway; 4 Department of Methods Development and Analytics, Norwegian Institute of Public Health, Oslo, Norway; 5 Department of Gastroenterology, Division of Medicine, Akershus University Hospital, Lørenskog, Norway; 6 Institute for Clinical Medicine, University of Oslo, Oslo, Norway; 7 Centre for Bioinformatics, Department of Pharmacy, University of Oslo, Oslo, Norway; 8 Department of Research, Cancer Registry of Norway, Oslo, Norway; 9 Department of Pathology, Akershus University Hospital, Lørenskog, Norway

**Keywords:** Colorectal cancer, Tumor biopsies, 16S rRNA amplicon sequencing, *Fusobacterium* species and subspecies, Biofilm bacteria

## Abstract

Accumulating evidence has related the gut microbiota to colorectal cancer (CRC). *Fusobacterium nucleatum* has repeatedly been linked to colorectal tumorigenesis. The aim of this study was to investigate microbial composition in different sampling sites, in order to profile the microbial dynamics with CRC progression. Further, we characterized the tumor-associated *F. nucleatum* subspecies. Here, we conducted Illumina Miseq next-generation sequencing of the 16S rRNA V4 region in biopsy samples, to investigate microbiota alterations in cancer patients, patients with adenomatous polyp, and healthy controls in Norway. Further, *Fusobacterium* positive tumor biopsies were subjected to MinION nanopore sequencing of *Fusobacterium*-specific amplicons to characterize the *Fusobacterium* species and subspecies. We found enrichment of oral biofilm-associated bacteria, *Fusobacterium*, *Gemella*, *Parvimonas*, *Granulicatella*, *Leptotrichia*, *Peptostreptococcus*, *Campylobacter*, *Selenomonas*, *Porphyromonas*, and *Prevotella* in cancer patients compared to adenomatous polyp patients and control patients. Higher abundance of amplicon sequence variants (ASVs) classified as *Phascolarctobacterium*, *Bacteroides vulgatus*, *Bacteroides plebeius*, *Bacteroides eggerthii*, *Tyzzerella*, *Desulfovibrio*, *Frisingicoccus*, *Eubacterium coprostanoligenes *group, and *Lachnospiraceae* were identified in cancer and adenomatous polyp patients compared to healthy controls. *F. nucleatum* ssp. *animalis* was the dominating subspecies. *F. nucleatum* ssp. *nucleatum*, *F. nucleatum* ssp. *vincentii*, *Fusobacterium pseudoperiodonticum*, *Fusobacterium necrophorum*, and *Fusobacterium gonidiaformans* were identified in five samples. Several biofilm-associated bacteria were enriched at multiple sites in cancer patients. Another group of bacteria was enriched in both cancer and polyps, suggesting that they may have a role in polyp development and possibly early stages of CRC.

**Supplementary Information:**

The online version contains supplementary material available at 10.1007/s10096-023-04551-7.

## Introduction

Colorectal cancer (CRC) is one of the most common cancers in Norway and worldwide. The incidence of CRC has increased significantly in recent years, and Norway now has one of the world’s highest incidences of colorectal cancer, with 4550 new cases reported in 2021 [1].

Over the last decade, emerging evidence suggests involvement of the gut microbiota in initiation and progression of CRC. Already in 2012, two independent groups showed significantly higher levels of the anaerobic oral commensal *Fusobacterium nucleatum* in tumor tissues compared to healthy tissues [2, 3]. Following this, *F*. *nucleatum* has repeatedly been reported in tumor samples and has also been associated with CRC cell proliferation, tumor invasion, and lymph node metastases [4–6].

Several studies have further shown that larger communities of pathogenic microorganisms are involved in progression of CRC, specifically through establishment of densely populated biofilms [7, 8]. Biofilms have been linked to CRC development through enhanced epithelial permeability, tissue inflammation, and bacterial invasion of the intestinal epithelium [9]. Bacteria that frequently have been associated with CRC biofilms are *Fusobacterium*, *Peptostreptococcus*, *Campylobacter*, *Parvimonas*, *Streptococcus*, and *Granulicatella*, among others [10–14]. The documented microbiota in CRC is not consistent between studies, apart from the repeated detection of *F. nucleatum*. Although *F. nucleatum* is clearly associated with CRC, very few studies have investigated different *F. nucleatum* subspecies associated with CRC [15–17]. *F. nucleatum* is a heterogeneous species, currently divided into four subspecies, *F. nucleatum* ssp. *nucleatum*, ssp. *animalis*, ssp. *vincentii*, *and* ssp. *polymorphum* [18]. It has been proposed that these are sufficiently divergent to be characterized as different species [19]. To gain a deeper understanding of *F. nucleatum* in CRC progression, it could be important to identify which subspecies are involved in CRC. Additionally, if *F. nucleatum* is to be utilized as a biomarker for CRC, identifying the different *F. nucleatum* subspecies is crucial.

The role of *F. nucleatum* as a driver of disease initiation or a passenger able to colonize or infect cancer tissues and contribute to disease progression has been debated [20]. Several studies argue that *F. nucleatum* appears at the tumor site after cancer development, attaching to Gal-GalNAc receptor that is overexpressed in tumor cells [21, 22]. Conversely, Tomkovich et al. showed that human bacterial biofilms present in non-neoplastic tissues were able to initiate CRC in a mouse model and suggested that bacterial biofilms could be important progenitors for CRC [23]. Most studies that have investigated the tumor microbiome composition have examined biopsies from the tumor sites and adjacent healthy tissues [8, 12]. Flemer et al. showed that a CRC-associated microbiota was found also in adjacent healthy tissues 2–30 cm away from the tumor and argued that a CRC distinctive microbiota was established prior to CRC development [24]. Unraveling the microbiota in larger parts of the colon could show if the changes indeed are local or widespread throughout the colon. Furthermore, patients with adenomatous polyps are valuable when studying CRC progression. Although only 5% of adenomatous polyps progress to cancerous tumors, most of the colorectal cancers evolve through adenomatous polyps, and the risk increases with polyp size [25, 26]. It is therefore important to examine the microbial composition in polyps to assess if there are any microbial signatures already at the polyp stage.

In the present study, we have characterized the mucosal microbiome in colonic biopsies from different sampling sites (ascending colon, tumor or polyp, adjacent healthy tissue, and colon sigmoideum) from subjects of cancer, adenomatous polyps, and controls through 16S rRNA amplicon sequencing. Furthermore, *Fusobacterium*-positive tumor biopsies were subjected to MinION nanopore sequencing to characterize the *Fusobacterium* subspecies. The aim of the study was to investigate microbial composition in different sampling sites, in order to profile the microbial dynamics with CRC progression.

## Materials and methods

### Study population and sampling

**Fig. 1:**
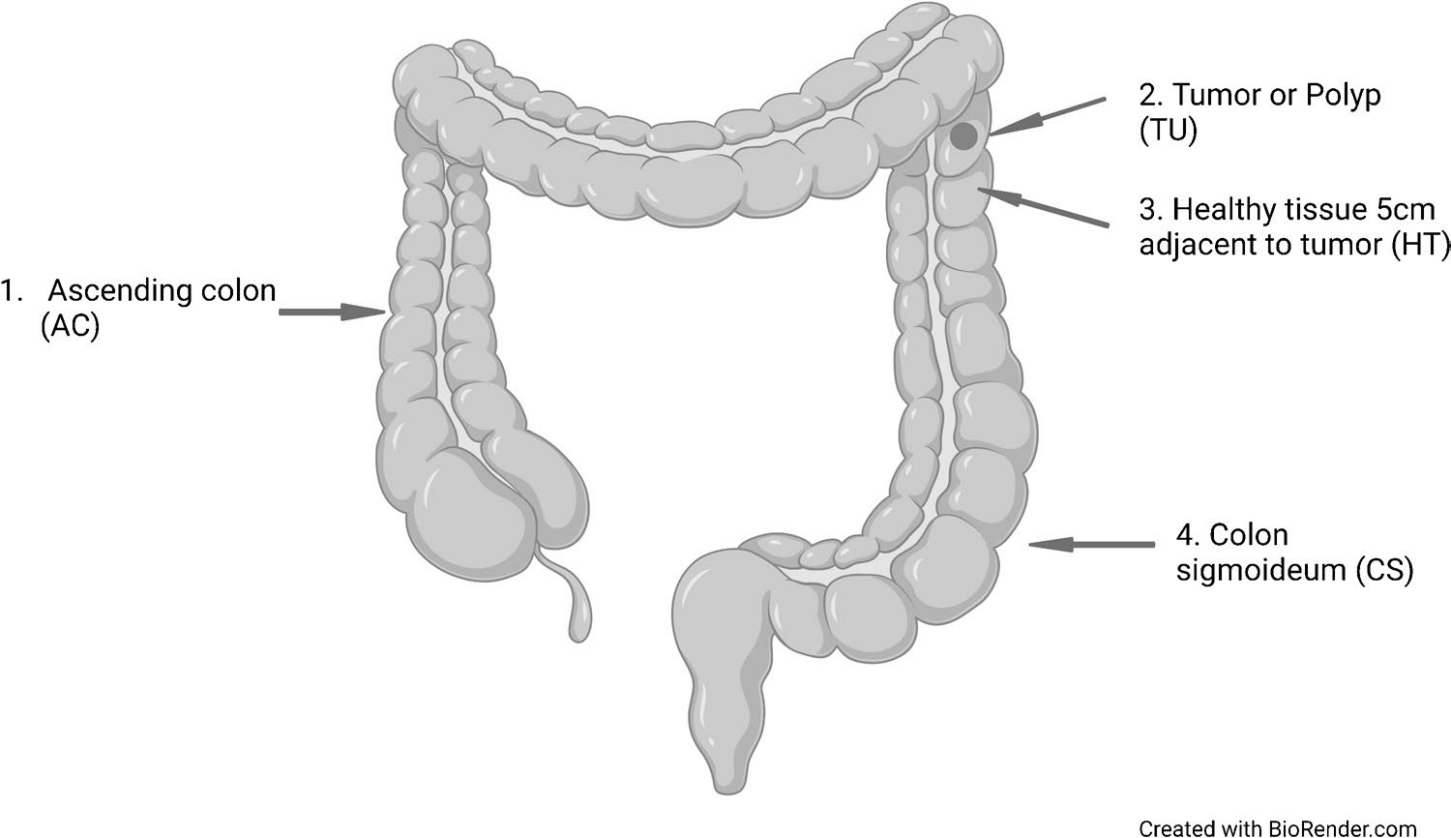
Illustration of the sampling sites where the biopsies were collected from this study

Seventy-two subjects who underwent colonoscopy at Akershus University Hospital from 2014 to 2017 were included in the present study. Prior to scheduled colonoscopy, participants were contacted and informed about the study. Written informed consent and questionnaire about age, gender, weight, height, diet, and smoking status were obtained from all the participants who were included in the study (Table 1). Participants were divided into three groups based on the findings during the colonoscopy, patients with CRC (*n* = 25), patients with adenomatous polyps (*n* = 25), and healthy controls (*n* = 22). Healthy controls had no lesion detected during colonoscopy. Colonic mucosal biopsies (sized 2–3 mm) from four positions (ascending colon, cancerous tissue or polyp, adjacent healthy tissue, and colon sigmoideum) were collected from patients with cancer and adenomatous polyps. From healthy controls, biopsies were collected from the ascending colon and colon sigmoideum (Fig. 1). All biopsies were stored in Allprotect Tissue Reagent (Qiagen, Hilden, Germany) immediately after collection, according to the manufacturer’s instructions.

**Table 1:** Demographic characteristics of the study group

Characteristics	Number of patients		
	Cancer (*n* = 25)	Polyp (*n* = 25)	Control (*n* = 22)
Age in years (average)	69.3	66.9	58.5
Sex (F/M)	7/18	14/11	9/13
Weight in kg (missing)	77.9 (3)	82.9 (4)	80.9 (2)
Smoking (yes/no/missing)	(2/20/3)	(2/21/2)	(2/18/2)
Vomiting or diarrhea (yes/no/missing)	(6/16/3)	(3/20/2)	(11/9/2)
Antibiotics (yes/no/missing)	(2/20/3)	(4/19/2)	(1/19/2)
Histologic diagnosis			
Adenocarcinoma	25	NA	NA
Location of tumor: *n* (%)			
Cecum	6 (24%)	NA	NA
Ascending colon	3 (12%)	NA	NA
Transverse colon	2 (8%)	NA	NA
Sigmoid colon	9 (36%)	NA	NA
Rectosigmoid junction	2 (8%)	NA	NA
Rectum	3 (12%)	NA	NA

### Extraction of DNA

DNA from 2/3 biopsies collected from all three study groups were extracted in 2016–2017 as previously reported [27]. DNA from the remaining biopsies were extracted in 2021 using the same protocol. Microbial DNA were purified using the AllPrep DNA/RNA Mini Kit (Qiagen) following a modified protocol published by Moen et al. [28]. In short, the biopsies were subjected to a thorough lysis and homogenization procedure, involving both enzymatic and mechanical lysis steps, before following the manufacturer`s instructions. The DNA was eluted using 40 µl nuclease-free water and was stored at − 20 °C. One extraction blank was included as negative control for each reagent lot to assess for DNA contamination. Extraction negative controls were run through the wet lab procedure, from the nucleic acid purification step through the sequencing process, to detect possible reagent contamination. Five positive control samples with different community structures were included. The concentration of DNA was assessed using NanoDrop 200 Spectrophotometer (Thermo Fisher Scientific, Waltham, MA, USA). The quality was obtained using the OD260/OD280 and OD260/OD230 ratios for purity assessment of the samples.

### 16S rRNA amplicon sequencing

**Fig. 2:**
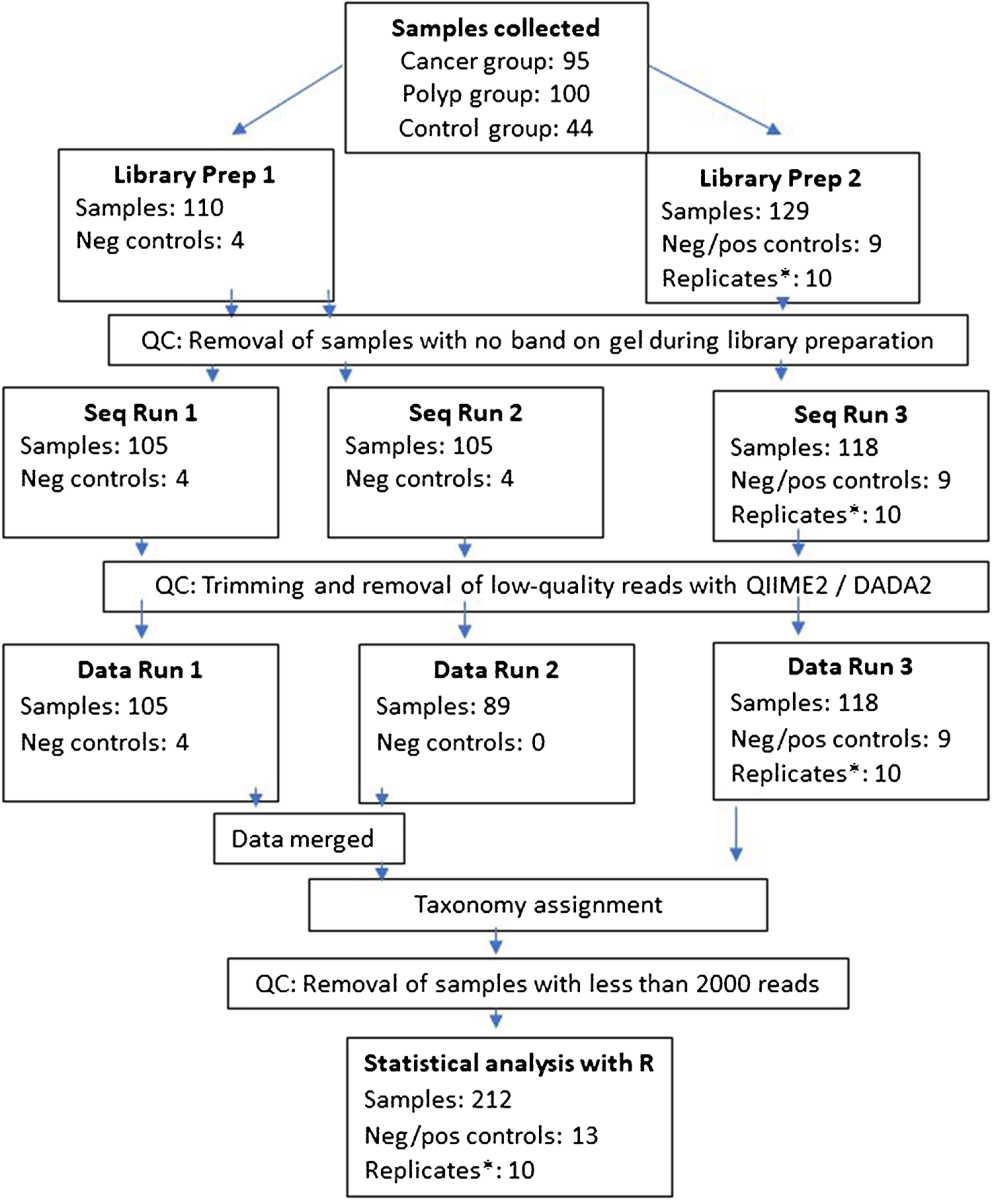
Schematic illustration of 16S rRNA amplicon sequencing workflow. Asterisk indicates that 10 samples from Library prep 1 were included in Library Prep 2 to control for batch effects due to run-to-run variation

A total of 239 samples from the three groups of participants (cancer = 25, polyp = 25, and control = 22) were analyzed for 16S rRNA amplicon sequencing (Fig. 2). PCR amplification of 16S rRNA V4 region was performed using 16S forward primer (16Sf V4: GTGCCAGCMGCCGCGGTAA) and 16S reverse primers (16Sr V4: GGACTACHVGGGTWTCTAAT) [29]. 16S rRNA gene amplification was performed in a total volume of 23 µl using 4 µl of 125 ng/µl DNA, 2 µl of paired set of index primers, and 17 µl of AccuPrime Pfx SuperMix (Thermo Fisher Scientific). PCR thermal cycling conditions and primer combinations are as described in the MiSeq Wet Lab SOP, except for the annealing temperature and the extension temperature being set to 50 and 68 °C, respectively (https://github.com/SchlossLab/MiSeq_WetLab_SOP/blob/master/MiSeq_WetLab_SOP.md). Access date 25 February 2022.

Amplicons were assessed using 1.5% agarose gel and pooled into three groups according to their band intensity. The samples that showed no band on gel (cancer = 4, polyp = 8, and healthy controls = 4) were not included in the NGS analysis. Pooled amplicon libraries around 400 bp were purified from 3% agarose gel using QIAquick Extraction Kit (Qiagen) and quantified using KAPA Library Quantification Kit (Universal) (Kapa Biosystems Inc., Wilmington, MA, USA). The sequencing was performed on the Illumina MiSeq platform (Illumina Inc., San Diego, CA, USA) using the MiSeq reagent kit v/2 according to the manufacturer’s instructions with addition of custom sequencing primers, index, and 8% PhiX (https://github.com/SchlossLab/MiSeq_WetLab_SOP/blob/master/MiSeq_WetLab_SOP.md). Access date 25 February 2022. Samples were sequenced in three runs in a consistent manner. One hundred five samples were sequenced paralleled in runs 1 and 2. One hundred twenty-eight samples were sequenced in run 3 (Fig. 2). To evaluate the reproducibility and variation of the three sequencing runs, ten samples (replicated samples) with both high and low intensity on agarose gel were included in all three sequencing runs. Extraction negative controls were included in all three runs. Five positive control samples were included in run 3.

### MinION sequencing

*Fusobacterium*-positive cancerous tumors from the cancer group (*n* = 18) were subsequently sequenced using *Fusobacterium*-specific targets to discriminate the species and subspecies of *Fusobacterium*. *Fusobacterium* zinc protease and 16S rRNA V3 region gene were amplified by PCR as described by Kim et al. and Walter et al. [30, 31], and amplicons were purified from 1% agarose gel using QIAquick Extraction Kit (Qiagen) prior to sequencing. One hundred fmol DNA was used for library construction using the protocol Amplicons by Ligation (SQK-LSK110) (Oxford Nanopore Technologies, Oxford, GB). Libraries from individual samples were loaded onto separate R9.4.1 Flongle Flow Cells (FLG-001), and sequencing was performed using the Oxford Nanopore Technologies (ONT) MinION MK1b device. Taxonomic classification was performed with the workflow “What’s in my pot” (WIMP) (Epi2me, Oxford Nanopore technologies) and the RefSeq sequence database (https://www.ncbi.nlm.nih.gov/refseq/) [32]. An internal quality control consisting of amplicons from lambda-phage DNA was included in each run. Positive controls consisting of amplicons from *F. nucleatum* ssp. *animalis* CCUG 32,879 and *F. nucleatum* ssp. *polymorphum* CCUG 9126 T, *F. nucleatum* ssp*. vincentii* CCUG 37843 T, and *F. nucleatum* ssp. *nucleatum* CCUG 33,059 were sequenced in separate experiments to assess sequencing fail rate. A negative control consisting of PCR grade water was run to assess microbial DNA contamination.

### Sequence analysis

MiSeq Reporter software (Illumina Inc.) was used for demultiplexing the reads, removing adapter and primer sequences, and for FASTQ file generation. The sequence data were processed using QIIME 2 (Quantitative Insights Into Microbial Ecology) version 2021.2.0. Raw FASTQ reads of the three MiSeq runs were quality filtered, trimmed, de-noised, and paired-end sequences merged, separately, using DADA2 [33] and the q2-dada2 plugin implemented in QIIME2. In brief, after inspection of the quality, sequences were trimmed to remove low-quality reads. For MiSeq runs 1 and 2, the last 50 bases from files R2 were trimmed off, keeping 250 and 200 bases for files R1 and R2, respectively. For MiSeq run. 3, 250 bases were kept for both files R1 and R2. This resulted in all sequences having a median Phred score of 27. Otherwise, default settings in the q2-dada2 plugin were used. Runs 1 and 2 were merged prior to further analysis as they contained the same samples. Taxonomy was assigned to the amplicon sequence variants (ASVs) using a pre-trained Native Bayes classifier, trained on the Silva V.138 reference sequence database and sequencing parameters used in the present study, via the q2-feature-classifier plugin.

### Statistical analysis

Sequences were taxonomically assigned to different taxa level to further analysis. Data analysis was performed in R (version 4.1.2) using packages phyloseq (version 1.36.0) and tidyverse (version 1.3.2).

To account for batch effect due to run-to run variation, we assessed the differences in overall microbiota community structure between the three sequencing runs using permutational multivariate analysis of variance (PERMANOVA) in R’s vegan package (version 2.5.7). We log-transformed the ASV counts and plotted bar plots to see if the feature counts are evenly distributed in the three sequencing runs.

**Fig. 3:**
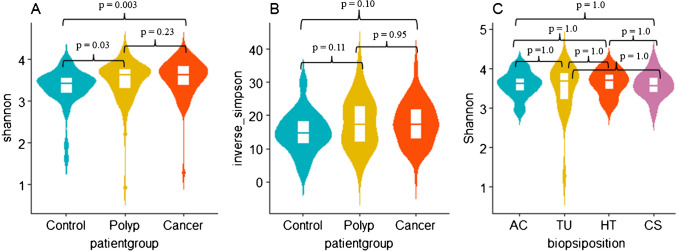
**A** Violin plots with box plots of Shannon bacterial alpha diversity index in mucosal biopsies from all sampling sites from all three groups. Pairwise Wilcoxon rank sum test revealed significant differences between cancer and healthy controls (*p* = 0.003) and polyp and healthy controls (*p* = 0.03). There is no significant difference between cancer and polyp (*p* = 0.23)). **B** Violin plots with box plots of Inverse Simpson diversity index in mucosal biopsies from all sampling sites all three groups. Pairwise Wilcoxon rank sum test revealed no significant differences between the groups (healthy controls vs polyp *p* = 0.11, healthy controls vs cancer *p* = 0.10, and polyp vs cancer *p* = 0.95). **C** Violin plots of Shannon diversity index in mucosal biopsies in cancer patients. Pairwise Wilcoxon rank sum test showed no significant differences between sampling sites in cancer patients. *p*-values are shown in Fig. 3C. AC, ascending colon; TU, tumor; HT, adjacent healthy tissue; CS, colon sigmoideum

To examine whether microbial composition differs across the samples and the groups, we conducted alpha and beta diversity analysis. We first calculated alpha diversity for all samples and assessed the differences in Shannon diversity and Inverse Simpson indices across the groups and within the groups, using Kruskal–Wallis rank sum test. Pairwise Wilcoxon rank sum test was conducted if there was a statistically significant difference between more than two groups. Violin plots were generated to visualize alpha diversity (ggplot2, version 3.3.5). Samples were rarefied to depth of 1000 ASVs prior to beta diversity analysis. Bray–Curtis distance matrix using principal coordinate analysis (PCoA) ordination (Fig. 3) was generated to compare the compositional changes of mucosal microbiota between the groups (vegan package, version 2.5.7). Further, we conducted PERMANOVA using ADONIS function to assess the differences in beta diversity between the groups. The analysis was based on Bray–Curtis dissimilarity with 999 permutations.

**Fig. 4:**
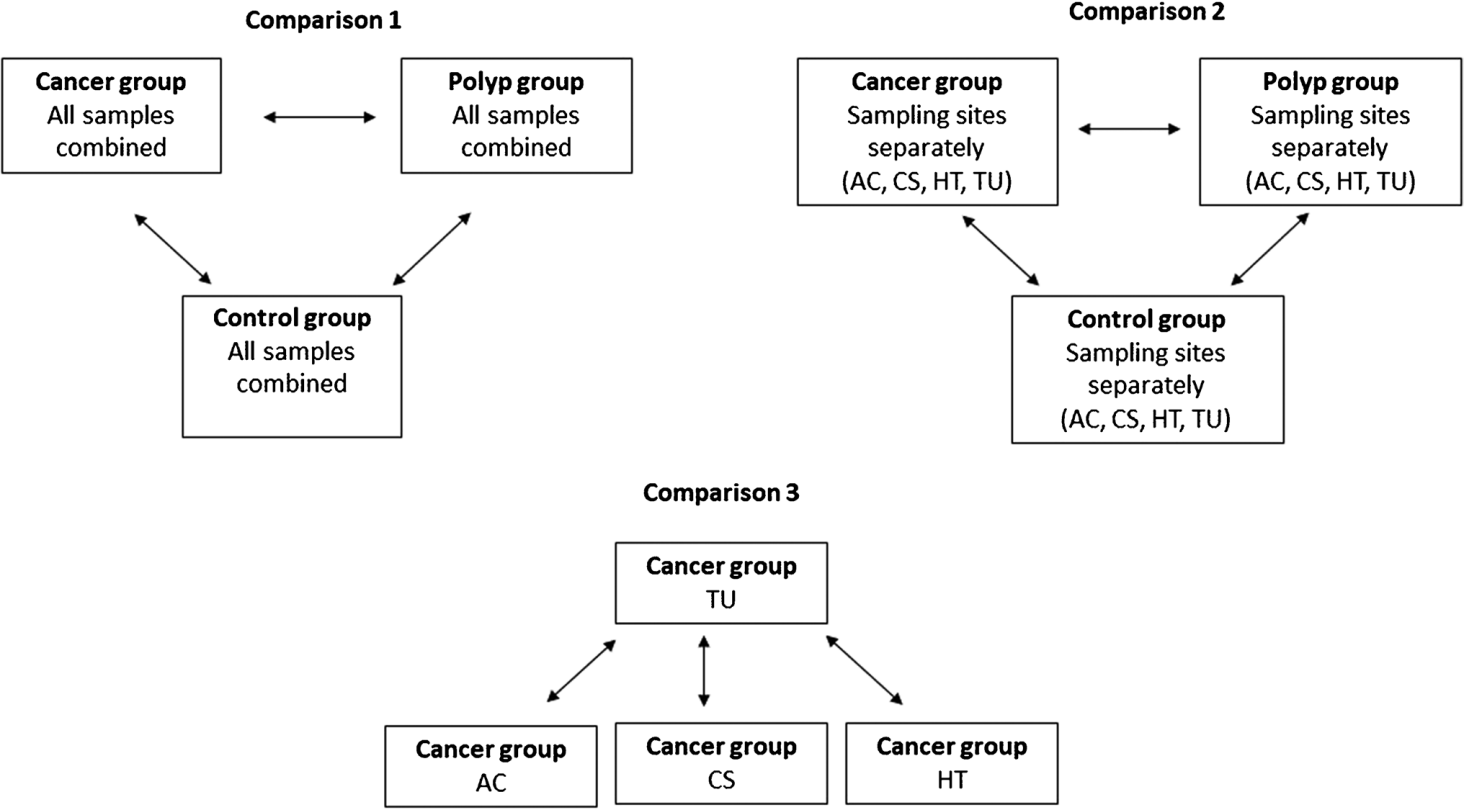
Illustration of comparisons performed in differential abundance analysis. AC, ascending colon; CS, colon sigmoideum; HT, adjacent healthy tissue; TU, tumor/polyp tissue. The results from differential analysis done in comparisons 1–3 are shown in Fig. 5 and Tables 2, 3, 4, and 5, respectively

DESeq2 (version 1.32.0) package was used to identify differentially abundant taxa between groups and between the sampling sites by pairwise comparison. ASVs with more than 10 reads in at least 10% of the samples were retained for differential abundance analysis. Figure 4 illustrates the comparisons that were performed. Due to small number of samples in this study, confounding factors such as, age, gender, vomiting, smoking, and antibiotic usage were not accounted for. *p*-value < 0.05 was considered significant for all statistical analyses.

**Table 2:** Significantly differentially abundant ASVs at specific sites in cancer group compared to adenomatous polyp group

Site	Family	Genus/species	baseMean	log2 fold change	padj
AC	*Prevotellaceae*	*Prevotella*	33.34	24.90	2.12E − 18
	*Lachnospiraceae*	*Family_Lachnospiraceae*_5	5.84	24.31	2.48E − 10
	*Clostridiaceae*	*Clostridium_sensu_stricto_1*	9.34	21.23	2.46E − 11
	*Oscillospiraceae*	*Colidextribacter*	13.84	− 6.94	1.90E − 3
	*Lachnospiraceae*	*Family_Lachnospiraceae*_4	120.20	− 9.11	2.07E − 03
	*Lachnospiraceae*	*Family_Lachnospiraceae*_6	66.63	− 9.84	1.02E − 02
	*Barnesiellaceae*	*Barnesiella*	44.04	− 22.51	1.52E − 08
	*Lachnospiraceae*	*Lachnoclostridium*	43.45	− 25.24	5.03E − 12
TU	*Prevotellaceae*	*Prevotella*	19.57	24.44	5.34E − 15
	*Peptostreptococcales-Tissierellales*	*Parvimonas*	16.94	24.24	1.08E − 16
	*Peptostreptococcaceae*	*Romboutsia*	10.75	23.59	2.35E − 20
	*Ruminococcaceae*	*CAG-352*	9.54	23.46	7.00E − 14
	*Bacteroidaceae*	*Bacteroides_ovatus*	5.28	22.66	4.47E − 13
	*Fusobacteriaceae*	*Fusobacterium*	593.42	9.82	3.27E − 06
	*Gemellaceae*	*Gemella*	152.93	5.46	3.64E − 03
	*Bacteroidaceae*	*Bacteroides_vulgatus*	4.89	− 22.54	5.02E − 13
	*Lachnospiraceae*	*Blautia*	7.38	− 23.11	1.41E − 13
	*Tannerellaceae*	*Parabacteroides*	22.62	− 24.68	3.20E − 15
HT	*Prevotellaceae*	*Prevotella*	14.20	24.57	1.56E − 15
	*Lachnospiraceae*	*Family_Lachnospiraceae*_5	8.59	23.90	2.40E − 18
	*Fusobacteriaceae*	*Fusobacterium*	50.88	8.31	6.81E − 04
	*Burkholderiaceae*	*Burkholderia-Caballeronia-Paraburkholderia*	19.16	− 7.52	4.33E − 06
	*Lachnospiraceae*	*Family_Lachnospiraceae*_6	9.68	− 23.08	1.20E − 13
	*Bacteroidaceae*	*Bacteroides_vulgatus*	22.19	− 23.68	3.27E − 14
	*Lachnospiraceae*	*Family_Lachnospiraceae*_4	26.83	− 24.51	4.03E − 18
	*Barnesiellaceae*	*Barnesiella*	34.34	− 24.85	1.56E − 15
CS	*Clostridiaceae*	*Clostridium_sensu_stricto_1*	4.03	20.12	8.83E − 08
	*Rhodanobacteraceae*	*Rhodanobacter*	23.75	3.17	4.28E − 02
	*Acidaminococcaceae*	*Phascolarctobacterium*	6.22	− 22.32	3.79E − 08

*AC* Ascending colon, *TU* tumor/polyp, *HT* adjacent healthy tissue, *CS* colon sigmoideum

**Table 3:** Significantly differentially abundant ASVs at specific sites in cancer group compared to healthy controls

Site	Family	Genus/species	baseMean	log2 fold change	padj
AC	*Acidaminococcaceae*	*Phascolarctobacterium*	123.30	26.33	3.58E − 21
	*Bacteroidaceae*	*Bacteroides_vulgatus*	40.89	24.35	1.87E − 09
	*Ruminococcaceae*	*Faecalibacterium*	6.64	23.35	2.14E − 09
	*Lachnospiraceae*	*Family_Lachnospiraceae*_5	5.84	22.48	1.17E − 08
	*Lachnospiraceae*	*Family_Lachnospiraceae*_1	34.93	21.78	3.18E − 09
	*Lachnospiraceae*	*Family_Lachnospiraceae*_2	10.70	19.34	3.94E − 06
	*Sutterellaceae*	*Sutterella*	129.12	11.02	4.49E − 03
	*[Eubacterium]_coprostanoligenes_group*	*[Eubacterium]_coprostanoligenes_group*	11.92	6.99	2.66E − 04
	*Oscillospiraceae*	*Colidextribacter*	13.84	− 7.33	5.49E − 04
	*Lachnospiraceae*	*Lachnoclostridium*	43.45	− 22.03	4.19E − 09
	*Barnesiellaceae*	*Barnesiella*	44.04	− 25.31	4.45E − 10
CS	*Acidaminococcaceae*	*Phascolarctobacterium*	156.42	26.49	1.60E − 18
	*Lachnospiraceae*	*Tyzzerella*	11.14	23.98	6.36E − 14
	*Bacteroidaceae*	*Bacteroides_plebeius*	10.49	23.57	4.82E − 09
	*Prevotellaceae*	*Prevotella*	7.04	20.55	6.50E − 07
	*Lachnospiraceae*	*Blautia*	6.61	19.07	6.05E − 06
	*Bacteroidaceae*	*Bacteroides_vulgatus*	11.45	18.56	1.07E − 05
	*Oscillospiraceae*	*UCG-005*	29.48	6.45	5.97E − 04
	*Oscillospiraceae*	*Colidextribacter*	12.62	− 6.39	1.90E − 02
	*Acidaminococcaceae*	*Phascolarctobacterium*	6.22	− 20.98	3.48E − 07

*AC* ascending colon, *CS* colon sigmoideum

**Table 4:** Significantly differentially abundant ASVs at specific sites in adenomatous polyp group compared to healthy controls

Site	Family	Genus/species	baseMean	log2 fold change	padj
AC	*Acidaminococcaceae*	*Phascolarctobacterium*	123.30	27.17	3.98E − 24
	*Lachnospiraceae*	*Family_Lachnospiraceae*_1	34.93	26.09	3.66E − 14
	*Bacteroidaceae*	*Bacteroides_vulgatus*	40.89	25.87	1.08E − 11
	*Lachnospiraceae*	*Family_Lachnospiraceae*_2	10.70	24.63	1.21E − 10
	*Ruminococcaceae*	*Faecalibacterium*	6.64	20.64	5.97E − 08
	*Sutterellaceae*	*Sutterella*	129.12	9.00	4.25E − 02
	*Bacteroidaceae*	*Bacteroides_eggerthii*	74.37	7.36	3.14E − 02
	*Burkholderiaceae*	*Burkholderia-Caballeronia-Paraburkholderia*	8.28	6.77	1.34E − 03
	*Rhodanobacteraceae*	*Rhodanobacter*	19.10	− 3.03	3.71E − 02
	*Prevotellaceae*	*Prevotella*	33.34	− 22.76	6.85E − 16
	*Clostridiaceae*	*Clostridium_sensu_stricto_1*	9.34	− 24.25	2.33E − 15
CS	*Acidaminococcaceae*	*Phascolarctobacterium*	156.42	27.60	7.41E − 19
	*Lachnospiraceae*	*Blautia*	6.61	24.10	5.78E − 09
	*Prevotellaceae*	*Prevotella*	7.04	23.96	5.95E − 09
	*Bacteroidaceae*	*Bacteroides_vulgatus*	11.45	23.59	9.47E − 09
	*Bacteroidaceae*	*Bacteroides_plebeius*	10.49	23.16	1.74E − 08
	*Lachnospiraceae*	*Tyzzerella*	11.14	21.81	1.27E − 10
	*Oscillospiraceae*	*UCG-005*	29.48	6.88	2.93E − 04
	*Rhodanobacteraceae*	*Rhodanobacter*	23.75	− 3.56	6.34E − 03
	*Clostridiaceae*	*Clostridium_sensu_stricto_1*	4.03	− 22.91	3.04E − 09

*AC* ascending colon, *CS* colon sigmoideum

**Table 5:** Significantly differentially abundant ASVs at different sites in cancer group

Position	Family	Genus/species	baseMean	log2 fold change	padj
TU vs HT	*Ruminococcaceae*	*Faecalibacterium*	10.89	23.54	5.84E − 08
	Fusobacteriaceae^*^	*Fusobacterium*	2145.87	5.74	7.65E − 02
	*Tannerellaceae*	*Parabacteroides*	15.97	− 21.48	6.30E − 07
TU vs AC	*Fusobacteriaceae*	*Fusobacterium*	2145.87	9.67	2.16E − 06
	*Tannerellaceae*	*Parabacteroides*	15.97	− 25.28	1.28E − 09
TU vs CS	*Lachnospiraceae*	*Blautia*	7.64	22.20	2.66E − 16
	*Fusobacteriaceae*	*Fusobacterium*	2145.87	8.23	1.05E − 04
	*Gemellaceae*	*Gemella*	96.03	5.06	9.70E − 03
	*Lachnospiraceae*	*Lachnoclostridium*	98.10	3.90	9.70E − 03
	*Tannerellaceae*	*Parabacteroides*	15.97	− 23.96	4.62E − 09

*AC* ascending colon, *TU* tumor/polyp, *HT* adjacent healthy tissue, *CS* colon sigmoideum

^*^Not significant

## Results

### Taxonomic analysis

From the 239 biopsy samples included in the present study, 223 (93%) passed all quality control steps from nucleic acid purification up to 16S rRNA sequencing (Fig. 2). Sequencing the V4 region of the 16SrRNA gene provided 5,567,904, 8,514,443, and 8,718,251 raw sequencing reads for sequencing runs 1, 2, and 3, respectively. Samples with less than 2000 counts (11 samples) were removed following DADA2 processing, resulting in a total of 212 samples and from 2005 to 532,329 high-quality reads per sample for runs 1 and 2 and from 3669 to 158,608 high-quality reads per sample for run 3, being eligible for further downstream analyses (Supplementary Table 1 A- C). Extraction negative controls gave less than 150 reads. Positive controls had on average 46,141 reads.

### Batch effect due to run-to-run variation

The effect of run-to-run variation on microbiome composition and number of reads was assessed using both the replicated samples (*n* = 6) and all samples. Differences in overall microbiota community between the three sequencing runs were assessed using PERMANOVA test. Results from PERMANOVA revealed that the microbial composition in the replicated samples did not differ significantly (*p* = 1) between the three runs (Supplementary Fig. 1). When considering all samples, there was a significant difference between the three runs (*p* = 0.001). The feature counts from samples from all three sequencing runs were evenly distributed (Supplementary Fig. 2), suggesting that the differences found in PERMANOVA was not due to the differences in feature counts.

### Shannon diversity differs between patient groups

We conducted the Kruskal–Wallis rank sum test to assess the differences in Shannon and Inverse Simpson diversity indices between the groups and within the groups. Shannon index showed statistically significant difference among groups (Kruskal–Wallis, *p* = 0.004). Further analysis with pairwise Wilcox test showed significant difference between cancer and healthy controls and polyps and healthy controls, *p* = 0.003 and *p* = 0.03, respectively (Fig. 3A). No statistically significant difference was found between cancer and polyp groups (*p* = 0.23). No statistically significant differences were observed in Inverse Simpson indices between the three groups (Kruskal–Wallis, *p* = 0.10, Fig. 3B). Comparing the microbiota of tumor and off-tumor tissues in cancer patients showed no statistically significant differences (Kruskal–Wallis, *p* = 0.96, Fig. 3C).

### Small but significant differences in microbial composition in biopsies from different patient groups

**Fig. 5:**
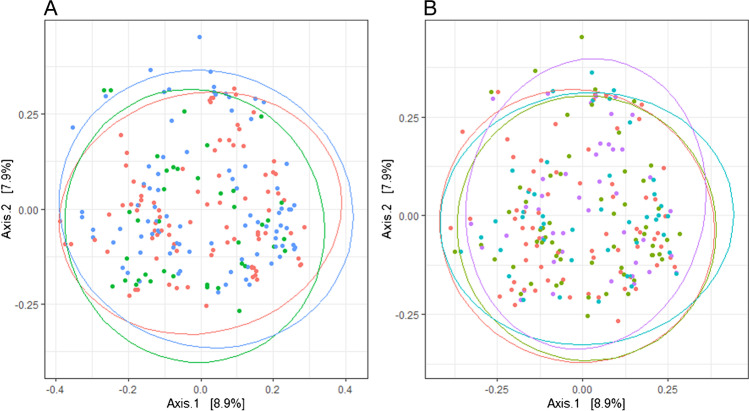
**A** Bray–Curtis bacterial beta diversity analysis of mucosal biopsies from all sampling sites from all three groups showed significant differences between the groups (*p* = 0.001); cancer patients (red), patients with adenomatous polyps (blue) and healthy controls (green). **B** Beta diversity analysis did not reveal any significant differences between mucosal biopsies of different sampling sites (*p* = 0.998); tumors and polyps (purple), adjacent healthy tissues (blue), colon sigmoideum (green) and ascending colon (red). Each point represents a biopsy

The microbial composition in the cancer group, adenomatous polyp group, and the healthy controls were compared using Bray–Curtis index principal coordinate analysis (Fig. 5) and PERMANOVA to assess whether they differ significantly from each other. Results from PERMANOVA revealed that the microbial composition in these three groups differ significantly (*p* = 0.001). However, only 2.3% (*R*^2^ = 0.023) of the variation is explained by this grouping. Comparing beta diversity of the microbial composition at the same biopsy site between different patient groups showed no significant differences (*p* > 0.05). No significant difference in the microbial composition was identified between the tumor tissues and the adjacent healthy tissues in the cancer group (*p* = 0.90).

### Comparison of taxa between patient groups demonstrates group specific bacteria

**Fig. 6:**
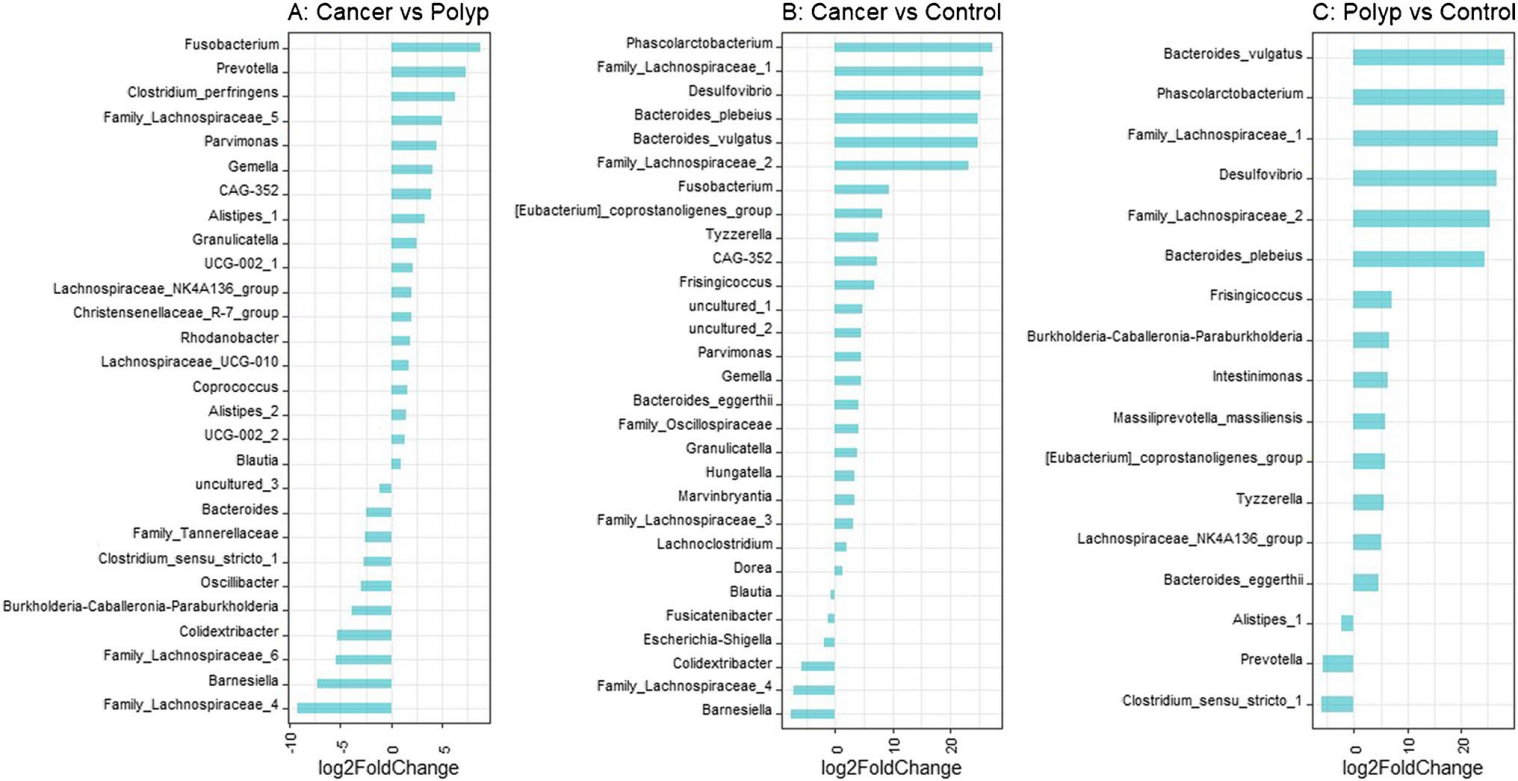
The figure illustrates enriched and depleted microbial ASVs between cancer, adenomatous polyp, and healthy controls (*p* < 0.05). **A** Cancer group vs adenomatous polyp group. **B** Cancer group vs healthy controls. **C** Adenomatous polyp group vs healthy controls. Abundance of *Fusobacterium*, *Gemella*, *Parvimonas*, and *Granulicatella* were higher in the cancer group compared to both other groups. ASVs classified as *Phascolarctobacterium*, *B. vulgatus*, *B. plebeius*, *B. eggerthii*, *Tyzzerella*, *Desulfovibrio*, *Frisingicoccus*, and *Eubacterium coprostanoligenes*_group and two ASVs classified as *Lachnospiraceae* were identified with higher abundance in both cancer and adenomatous polyp groups compared to healthy controls

Comparison of abundance of ASVs between the cancer patients (87 samples), patients with adenomatous polyps (86 samples) and healthy controls (39 samples) revealed significant differences between the groups (Fig. 6A–C). Differential analysis between the cancer group and the adenomatous polyp group demonstrated statistically significant difference in twenty-eight ASVs, of which eighteen were higher in abundance in the cancer group. Comparison between the cancer group and healthy controls, and between the adenomatous polyp group and healthy controls demonstrated 29 and 17 significantly different ASVs, respectively.

We found that ASVs classified as *Phascolarctobacterium*, *Bacteroides vulgatus*, *Bacteroides plebeius*, *Bacteroides eggerthii*, *Tyzzerella, Desulfovibrio*, *Frisingicoccus*, and *Eubacterium coprostanoligenes*_group and two ASVs classified as *Lachnospiraceae* were increased in both cancer and adenomatous polyp groups compared to healthy controls (Fig. 6A and B). Four bacterial taxa were found to be higher in abundance in the cancer group compared to the adenomatous polyp group and the healthy controls; these were *Fusobacterium*, *Gemella*, *Parvimonas*, and *Granulicatella.* All significantly abundant taxa are presented in Fig. 6 and Supplementary Table 2A – C.

### Comparisons of taxa at individual sampling sites between patient groups demonstrate site-specific differences

We compared ASVs at different sampling sites (ascending colon, tumor/polyp, adjacent healthy tissue, and colon sigmoideum) between cancer, adenomatous polyp, and healthy controls to see whether we could find any site-specific differences (Fig. 4). Comparison of sites between cancer and polyp groups showed that most of the differences we identified were found at tumor/polyp site, adjacent healthy tissue, and ascending colon (Table 2). Highest fold changes were identified at the tumor site compared to the polyp site, especially for the biofilm-associated bacteria; *Parvimonas*, *Prevotella*, *Fusobacterium*, and *Gemella*. We found that *Parabacteroides* was lower in abundance at the tumor site in the cancer group compared to the polyp site in the polyp group (log2 fold change =  − 24.68,). *Prevotella* was significantly higher in abundance at all sites, except colon sigmoideum in the cancer group compared to the polyp group. We found significantly higher abundance of *Sutterella* and *Faecalibacterium* at ascending colon in the cancer and polyp groups compared to controls when we compared individual sites (Tables 3 and 4).

### Comparison between tumor site and off-tumor sites in cancer patients demonstrates enrichment of Fusobacterium and lower abundance of Parabacteroides

We compared the microbial composition in biopsies taken from different sites in cancer patients. *Parabacteroides* was significantly lower in abundance at the tumor site compared to adjacent healthy tissue, ascending colon, and colon sigmoideum. Abundance of *Fusobacterium* was significantly higher at the tumor site compared to ascending colon and colon sigmoideum. Comparison between the tumor site and adjacent healthy tissue showed fivefold higher abundance of *Fusobacterium* at the tumor site, although the result was not significant.

### Comparison of microbial dynamics at the taxonomic genus level

Differential abundance of bacterial taxa between the patient groups were also compared at the genus level. *Fusobacterium*, *Gemella*, *Parvimonas*, and *Granulicatella* were still significantly increased in the cancer group compared to both other groups. Additionally, several other biofilm-related genera were found to be increased in the cancer group compared to both the polyp group and the control group; these were *Leptotrichia*, *Peptostreptococcus*, *Campylobacter*, *Porphyromonas, Selenomonas* and *Prevotella.* Our results demonstrate that two or more of these biofilm-associated bacteria appear together in nearly all tumors. This co-occurrence was also seen in some polyps samples (Supplementary Fig. 3).

**Fig. 7:**
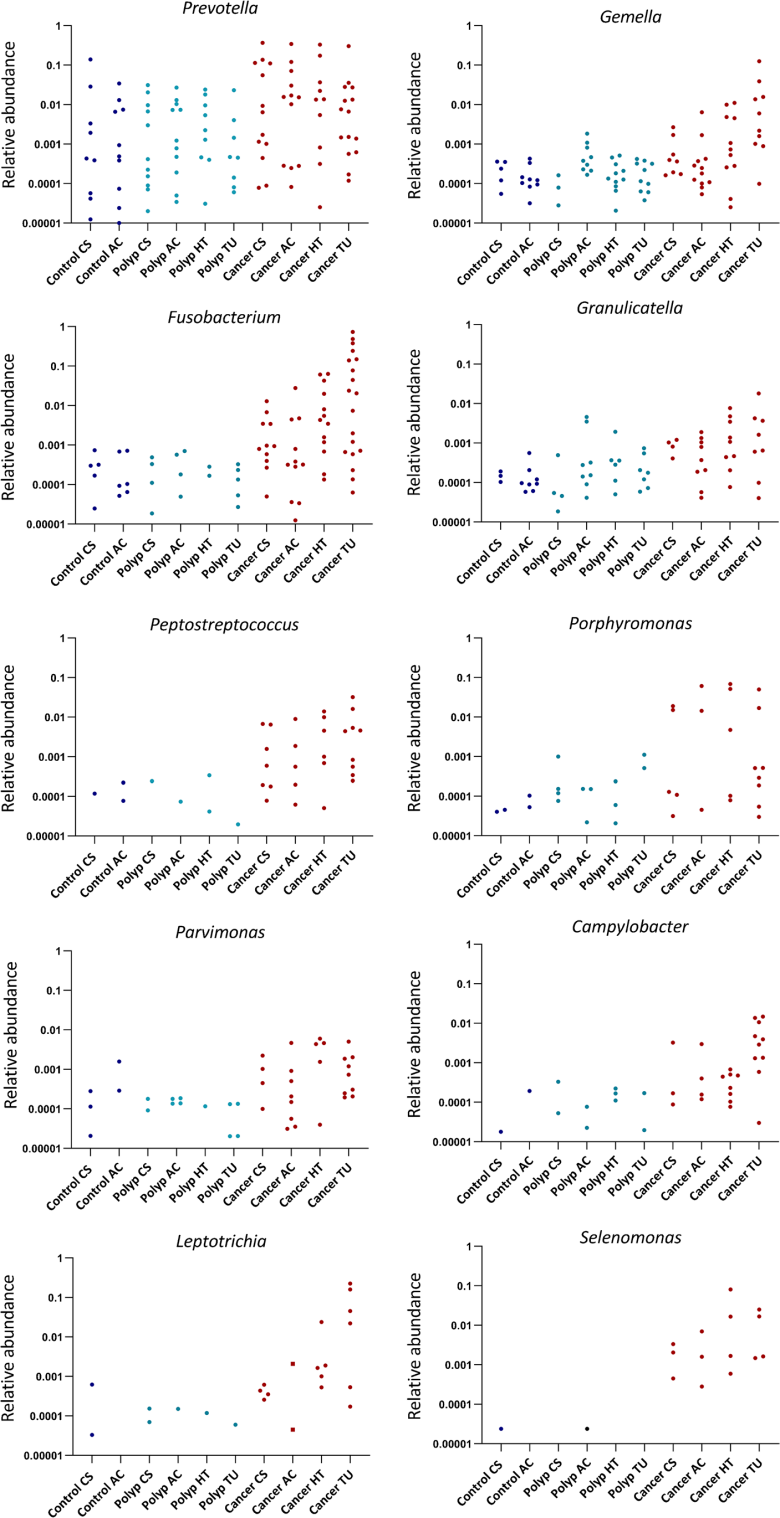

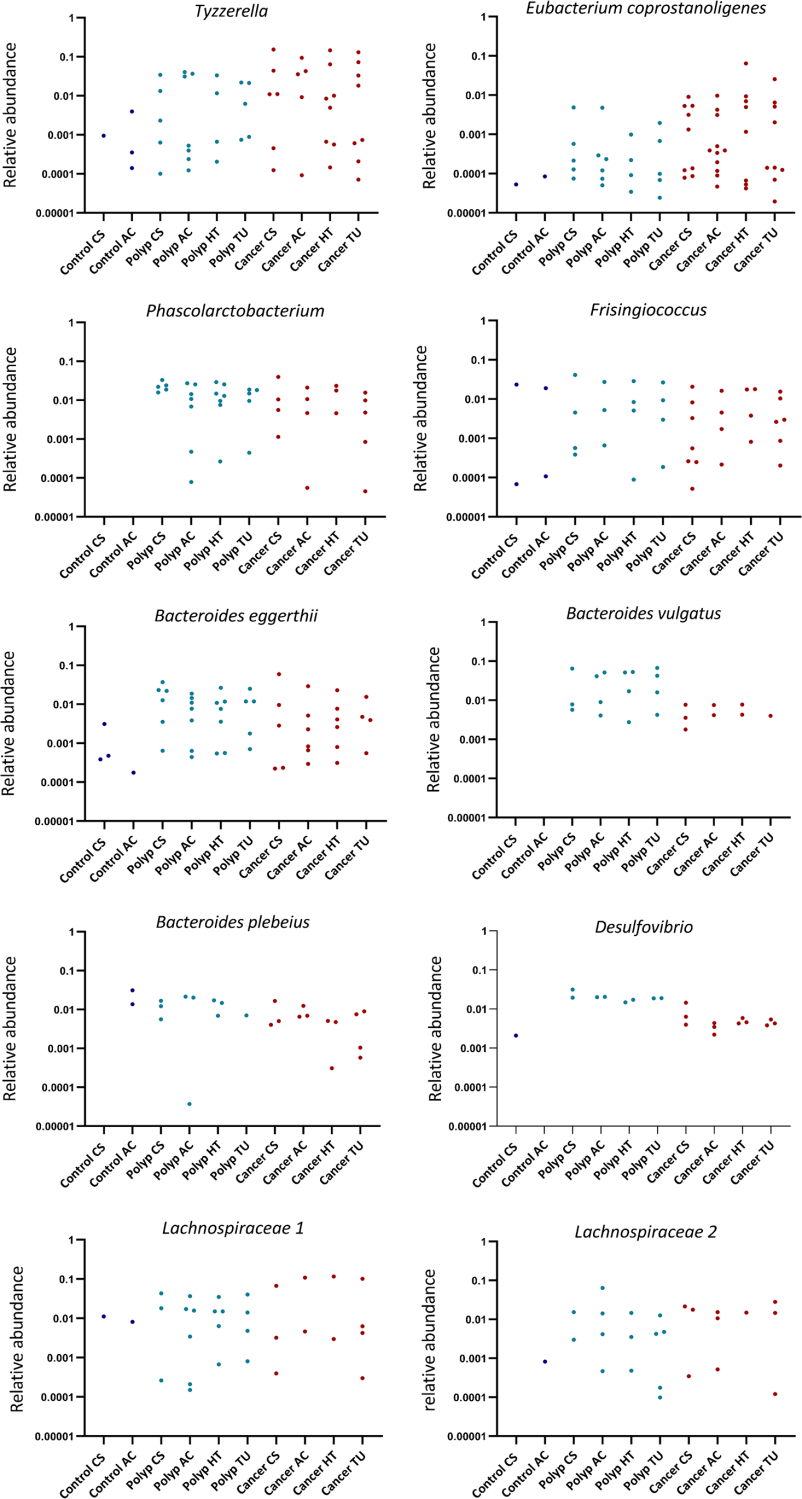
**A** The dynamics of microbial taxa with CRC progression shown for genera that were significantly increased in cancer compared to polyps and controls. The figure shows the relative abundance of taxa that were present at different samplings sites in cancer, polyps, and controls. **B** The dynamics of microbial taxa with CRC progression shown for ASVs that were significantly increased both in cancer and polyps compared to controls. The figure shows the relative abundance of taxa at different sampling sites in cancer, polyps, and controls

Many of the observed differences in ASVs between cancer and polyps compared to controls were no longer significant at genus level. *Tyzzerella* was only significantly increased between cancer and controls when compared at the genus level. However, *Butyrivibrio* appeared as significantly different between both cancer and controls and polyps and controls. All results are shown in Supplementary Table 3 A – C. Figure 7 illustrates the microbial dynamics with CRC progression.

### Discrimination of Fusobacterium nucleatum subspecies in tumor biopsies from cancer patients

**Fig. 8:**
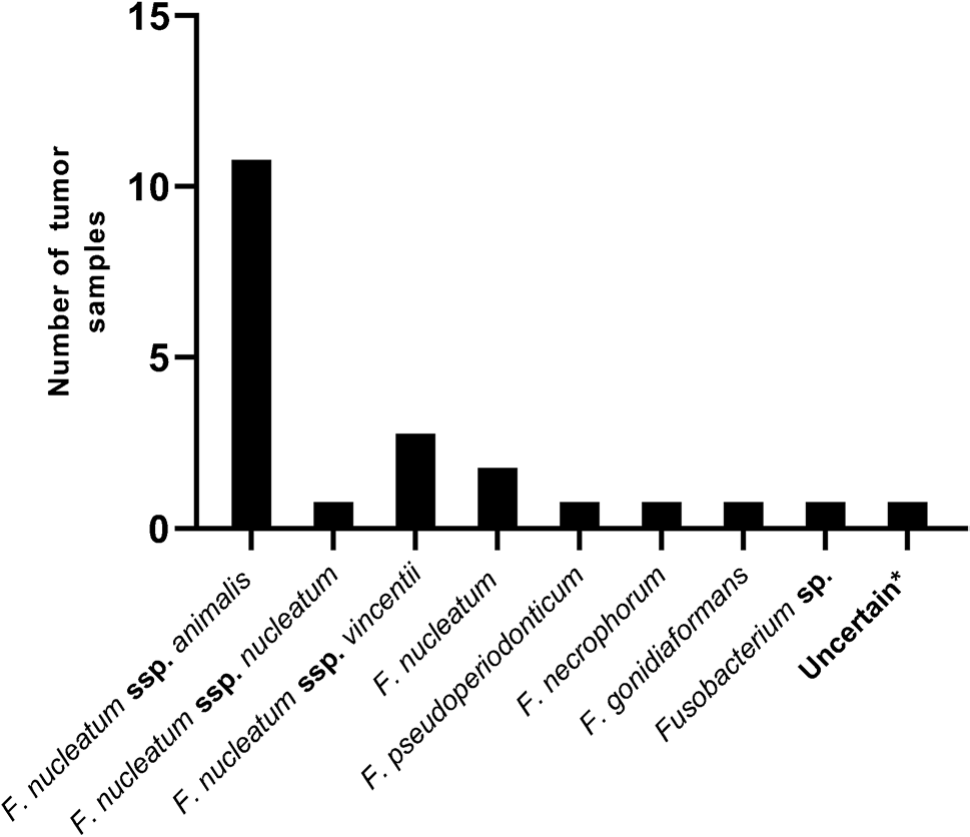
Bar chart illustrates the distribution of *Fusobacterium* species and subspecies in the colorectal cancer tumors. Results were obtained using *Fusobacterium* specific amplicon sequencing with MinION and taxonomic classification by WIMP. Asterisk indicates uncertain results due to low sequencing depth

MinION sequencing followed by taxonomic identification by WIMP identified *Fusobacterium* specific sequences for 18 *Fusobacterium* positive tumor samples (Fig. 8 and Supplemental Table 4). Results from samples with less than 100 reads from MinION were reported as uncertain. *F. nucleatum* ssp. *animalis* was the dominating subspecies identified in 11 *Fusobacterium* positive tumors. In five samples, *F. nucleatum* ssp. *nucleatum*, *F. nucleatum* ssp. *vincentii*, *F. pseudoperiodonticum*, *F. necrophorum*, and *F. gonidiaformans* were identified, respectively. Quality controls consisting of reference strains from the four different subspecies illustrated that all subspecies were correctly identified (Supplemental Table 4). However, taxonomic analysis of *F. nucleatum* ssp. *animalis* showed that 4% of the *Fusobacterium* reads were wrongly classified as ssp. *vincentii* (Supplementary Table 4). Therefore, read classifications with lower abundance than 10% of the total number of *Fusobacterium* reads were not considered.

## Discussion

Colorectal cancer is a heterogeneous disease associated with the environmental factors such as high-fat diet, obesity, lifestyle, and composition of the gut microbiome [34–37]. The present study was initiated to explore the microbial composition at different sampling sites of the colon from subjects of cancer, adenomatous polyps, and controls in a small but well-defined Norwegian cohort.

Our study showed higher alpha diversity in the cancer group and adenomatous polyp group compared to healthy controls, suggesting that these two groups consist of more diverse and evenly present microbiota than the healthy controls. Zhao et al. reported that patients with more oral-related microbiota had lower alpha diversity [38]. However, we could not see any association between oral-related microbiota and alpha diversity in our study. Studies are conflicting when reporting the relationship between gut microbiota in biopsies and alpha diversity. Thomas et al. demonstrated higher species diversity in rectal biopsy samples from cancer patients compared to non-cancer controls [39]. Microbial diversity in fecal samples has both been either reported to be decreased in CRC patients compared to healthy controls [40, 41] or to show no differences between controls and cancer patients [17]. It is worth noting that, although most of the studies use 16S rRNA gene sequencing for gut microbiome analysis, there are differences in the choice of the target variable region, sequencing platform used, and databases used for taxonomic assignment. In addition, low sequencing depth can lead to loss of rare species [42]. These factors may have led to sequencing bias and the differences in alpha diversity.

Beta diversity analyses revealed significantly different bacterial compositions between the patient groups in our study. Comparison of microbial composition in the cancer, adenomatous polyp, and control groups illustrates that some taxa appear to be specific to the cancer group, while others are increased in both cancer and polyp patients. Higher abundance of *Phascolarctobacterium*, *B. vulgatus*, *B. plebeius*, *B. eggerthii*, *Tyzzerella*, *Desulfovibrio*, *Eubacterium coprostanoligenes*, and *Frisingicoccus* in cancer patients and patients with adenomatous polyps compared to healthy controls may suggest that these taxa could provide a favorable environment for polyp formation and initial stages of cancer. Other studies that examined tumor-associated bacterial taxa also reported elevated abundances of *Phascolarctobacterium*, *Desulfovibrio*, and *Bacteroides* in cancer patients compared to healthy controls. *Desulfovibrio*, a Gram-negative sulfate-reducing bacterium, may contribute to mucosal inflammation through hydrogen sulfide production [43]. Toxin-producing strains of *Bacteroides fragilis* have often been associated with CRC and even suggested as a keystone pathogen for CRC development [39, 44]. In the present study, three other *Bacteroides* species were increased in polyps and cancers compared to healthy controls. *B. vulgatus* has been shown to decrease in abundance after CRC surgery and could potentially have a role in CRC [45]. Yachida et al. investigated the tumor microbiome in different stages of CRC and showed that *Phascolarctobacterium* was increased in early stages of colorectal disease [46]. Its role in disease development has yet to be described. *Phascolarctobacterium* is a Gram-negative anaerobic bacterium known to produce the short-chain fatty acid propionate and may therefore have beneficial effects for the intestinal mucosa [44]. *Eubacterium coprostanoligenes* was also significantly increased in polyps and cancers compared to controls in this study. This Gram-positive anaerobic bacterium has cholesterol-reducing properties [47]. To our knowledge, this species has not previously been associated with polyps or CRC; however, its close relative *Eubacterium rectale* has been described as a “driver” bacterium for CRC initiation by promoting colitis [48]. Further studies on species level are required to determine the role of these bacteria in CRC progression.

*Fusobacterium*, *Parvimonas*, *Gemella*, *Granulicatella*, *Leptotrichia*, *Peptostreptococcus*, *Campylobacter*, *Porphyromonas*, *Selenomonas*, and *Prevotella* were enriched in cancer patients compared to patients with adenomatous polyps and controls, suggesting they either have a role in cancer development or are favored in the cancerous state. Although higher in abundance in cancers, *Prevotella* were present in relatively high quantities at all sample sites in all patient groups. These Gram-negative anaerobic commensals are known to colonize several mucosal sites in the human body. *Prevotella* target peptides and amino acids for their digestion, resulting in ammonia production which would raise the local pH and neutralize hostile and acidic environment pH. This could facilitate the establishment of a more acid-intolerant bacterial flora [49], and an increase of *Prevotella* could possibly be important to favor a microbiota with oncogenic properties. Furthermore, *P. intermedia* has been shown to enhance the migration and invasion of CRC cells in conjunction with *F. nucleatum* [50] and could therefore be important in advanced stages of CRC.

The nine additional taxa that were significantly increased in cancer patients were all bacteria that are normally part of the oral microbiota. Biofilm-associated bacteria of oral origin are involved in many extra-oral diseases and have recently been associated with colorectal cancer [7, 14, 51]. Warren et al. identified co-occurrence of *Fusobacterium*, *Leptotrichia*, and *Campylobacter* in tumor samples, whereas Flemer et al. found several operational taxonomic units (OTUs) related to oral cavity bacteria in a subset of cancer samples, namely, *Fusobacterium*, *Porphyromonas*, *Anaerococcus*, *Prevotella*, *Granulicatella*, and *Parvimonas* [24, 51]. CRC biofilms do not necessarily consist of the same species in every patient, but it may appear that multispecies communities are required for invasion of tumor cells [7]. CRC biofilms have been described as essential for disruption of the colonic epithelial barrier [9, 52].

Comparison of the taxa present at different sampling sites in cancer patients showed that of the biofilm-associated genera, only *Fusobacterium* and *Gemella* were significantly different at the tumor site. This could suggest that the growth of *Fusobacterium* and *Gemella* is particularly favored in colorectal tumors. *Fusobacterium* is known for its cancer-promoting effect mediated by its virulence factor FadA. FadA is a adhesion protein that interacts with E-cadherin expressed on the epithelial cells resulting in activation of the β-catenin signaling pathway [5]. *Gemella* are Gram-positive facultative anaerobic cocci described as early colonizers of biofilms and have been associated with extra-oral opportunistic infections such as endocarditis. Case reports have described an association of *Gemella* endocarditis with underlying colonic malignancy [53, 54]. *G. morbillorum* was shown to be a promising biomarker for CRC [55]. Although significantly higher in tumor tissues, *Fusobacterium* and *Gemella* were also identified at non-neoplastic sites. The remaining biofilm-related bacterial taxa were present in similar quantities at all sites. This observation was also done by Flemer et al. and Dejea et al. who showed that the biofilm-associated bacteria were not restricted to cancerous tissues [9, 24]. Our analyses were extended to larger parts of the colon and illustrate that the taxa are present at all sampling sites. This supports the idea that the establishment of a cancer-associated microbiota appears prior to tumor development.

The anaerobic Gram-positive and opportunistic pathogen *Parvimonas micra* has recently been shown to promote CRC tumorigenesis in multiple mouse models. The pro-tumorigenic effect of *P. micra* was associated with altered immune responses and enhanced secretion of inflammatory cytokines in the gut [56]. *P. micra* has already been suggested as a non-invasive biomarker for colorectal cancer in a number of studies, including a Swedish cohort, closely related to the Norwegian population [57]. We found that the biofilm-associated taxa were not significantly different in adenomatous polyp patients compared to healthy controls. However, inspection of taxonomy data showed the presence of one or more of the biofilm-associated taxa in some polyp samples at several sampling sites. This may suggest a beginning of CRC-associated biofilm formation and that these polyps could potentially develop into tumors if the above-mentioned taxa have cancer promoting properties.

On the other hand, we cannot rule out that contamination between samples during colonoscopy may contribute to this finding. During colonoscopy, samples were first taken from ascending colon, then from tumor, adjacent tissue, and finally from colon sigmoideum. However, the findings of oral biofilm-associated taxa in the ascending colon strengthen the validity of our results.

We identified lower abundance of *Parabacteroides* at the tumor site compared to off-tumor sites in the cancer group. A lower abundance of *Parabacteroides* at tumor sites suggests that these bacteria succumb in the inflamed microenvironment. Similar to our findings, other studies report a depletion of *Parabacteroides* in inflammatory bowel disease patients compared to control patients or in inflamed tissue compared to uninflamed tissue [58, 59].

In the present study, *Fusobacterium* was detected in 21 of 25 tumor biopsies from cancer patients, confirming that *Fusobacterium* is associated with CRC. We identified *F. nucleatum* spp. *animalis* as the most common subspecies present in tumor tissues. This is in line with Ye et al. [15] and Bi et al. [60], who both found *F. nucleatum* ssp. *animalis* to be the dominating subspecies on colorectal tumors. In a review of oral bacteria in extra-oral infections, Han and Wang showed that selected subtypes of a given species, such as *F. nucleatum* ssp. *animalis*, are more prone to extra-oral translocations [61]. It is not known if this subtype possesses specific virulence factors or oncogenic properties that are not present in the other subspecies. The gene encoding the well-known adhesion protein FadA is present in all *F. nucleatum* subspecies, as well as other oral *Fusobacterium* spp. [62]. It is unknown if expression of the protein in different strains and environments could potentially differ. A possible explanation for the dominance of subspecies *animalis* on colorectal tumors may simply be overabundance in the oral cavity of cancer patients, but studies have shown that all four subspecies are present in the oral cavity of healthy individuals [63]. Although subspecies *animalis* was the most prevalent subspecies in the present study, an important finding was that five cancer patient samples contained other *F. nucleatum* subspecies and even other *Fusobacterium* species: *F. pseudoperiodonticum*, *F. necrophorum*, and *F. gonidiaformans*. This illustrates that a CRC biomarker assay targeting *Fusobacterium* should be able to detect additional *Fusobacterium* species. Large variations in detection rates of *Fusobacterium* spp. have been reported in different studies, ranging from 9 to 100% [64]. Both methodological and biological variations could explain these differences. In our previous study using qPCR with *NusG* as a qPCR target, we detected *Fusobacterium nucleatum* in 13 of 21 tumors, illustrating that some *Fusobacterium*-positive samples were not detected by the qPCR assay [27] and showing clearly a need for design of PCR assays capable of detecting of several *Fusobacterium* species.

We are aware of the limitations of this study such as small number of patients in each group and none accounting for confounders. Considering run-to-run variation, runs 1 and 2 contained the same samples, while run 3 had new samples; this may contribute to the difference shown in the diversity analysis between the three runs.

In conclusion, we have demonstrated enrichment of several bacteria with CRC progression. In particular, several biofilm-associated bacteria were increased in cancer patients. These are promising targets for detection of colorectal cancer. Another group of bacteria was enriched in both cancer and polyps, suggesting that they may have a role in polyp formation and initial stages of CRC. Although *F. nucleatum* ssp. *animalis* was the most prevalent in this study, other *F. nucleatum* subspecies and *Fusobacterium* species were detected. This illustrates that a pan-*Fusobacterium* PCR assay should be considered for detection of colorectal cancer. Further investigation and confirmation of these findings in a larger population will make it possible to develop microbial-based diagnosis for CRC.

## Supplementary Information

Below is the link to the electronic supplementary material.

Supplementary file1 (DOCX 108 KB)

Supplementary file2 (DOCX 57.1 KB)

## Data Availability

The data analyzed during the current study are not publicly available due to possible human DNA contaminations but may be made available upon reasonable request to the corresponding author.

## Code availability

The code may be made available upon written request to the corresponding author.
