# Supplementary material for: Microbial dynamics with CRC progression: a study of the mucosal microbiota at multiple sites in cancers, adenomatous polyps, and healthy controls
Source: Eur J Clin Microbiol Infect Dis. 2023 Jan 27;42(3):305–22. doi: 10.1007/s10096-023-04551-7 (PMC9899194; doi:10.1007/s10096-023-04551-7)
Supplement: Supplementary file 1 — Supplementary file1 (DOCX 108 KB) [file 10096_2023_4551_MOESM1_ESM.docx]

Supplementary Figure

**Microbial dynamics with CRC progression: a study of the mucosal microbiota at multiple sites in cancers, adenomatous polyps, and healthy controls**

Thulasika Senthakumaran^1^, Aina E. F. Moen^2,3,4^, Tone M. Tannæs^2,3^, Alexander Endres^5^, Stephan A. Brackmann^5,6^, Trine B. Rounge^7^,^8^, Vahid Bemanian^9^, Hege S. Tunsjø^1^

^1^Department of Life Sciences and Health, Oslo Metropolitan University, Oslo, Norway; ^2^Section for Clinical Molecular Biology (EpiGen), Akershus University Hospital, Lørenskog, Norway; ^3^Department of Clinical Molecular Biology, Institute of Clinical Medicine, University of Oslo, Oslo, Norway; ^4^Department of Methods Development and Analytics, Norwegian Institute of Public Health, Oslo, Norway; ^5^Department of Gastroenterology, Division of Medicine, Akershus University Hospital, Lørenskog, Norway; ^6^Institute for Clinical Medicine, University of Oslo, Oslo, Norway; ^7^Centre for Bioinformatics, Department of Pharmacy, University of Oslo, Oslo, Norway; ^8^Department of Research, Cancer Registry of Norway, Oslo, Norway; ^9^Department of Pathology, Akershus University Hospital, Lørenskog, Norway

Supplementary Figure 1


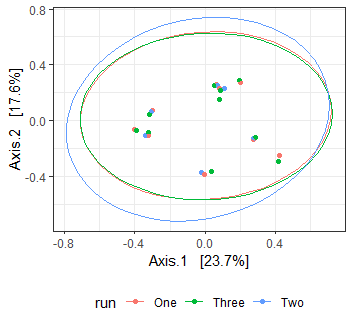


Supplementary Figure 1: Bray-Curtis bacterial diversity of replicated samples showed not significantly difference between the three runs.

Supplementary Figure 2


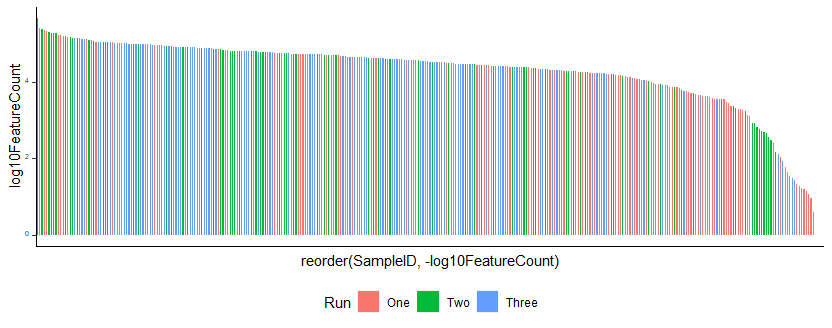


Supplementary Figure 2: Number of reads (Feature counts) transformed to log 10 from samples from all three sequencing runs.

Supplementary Figure 3


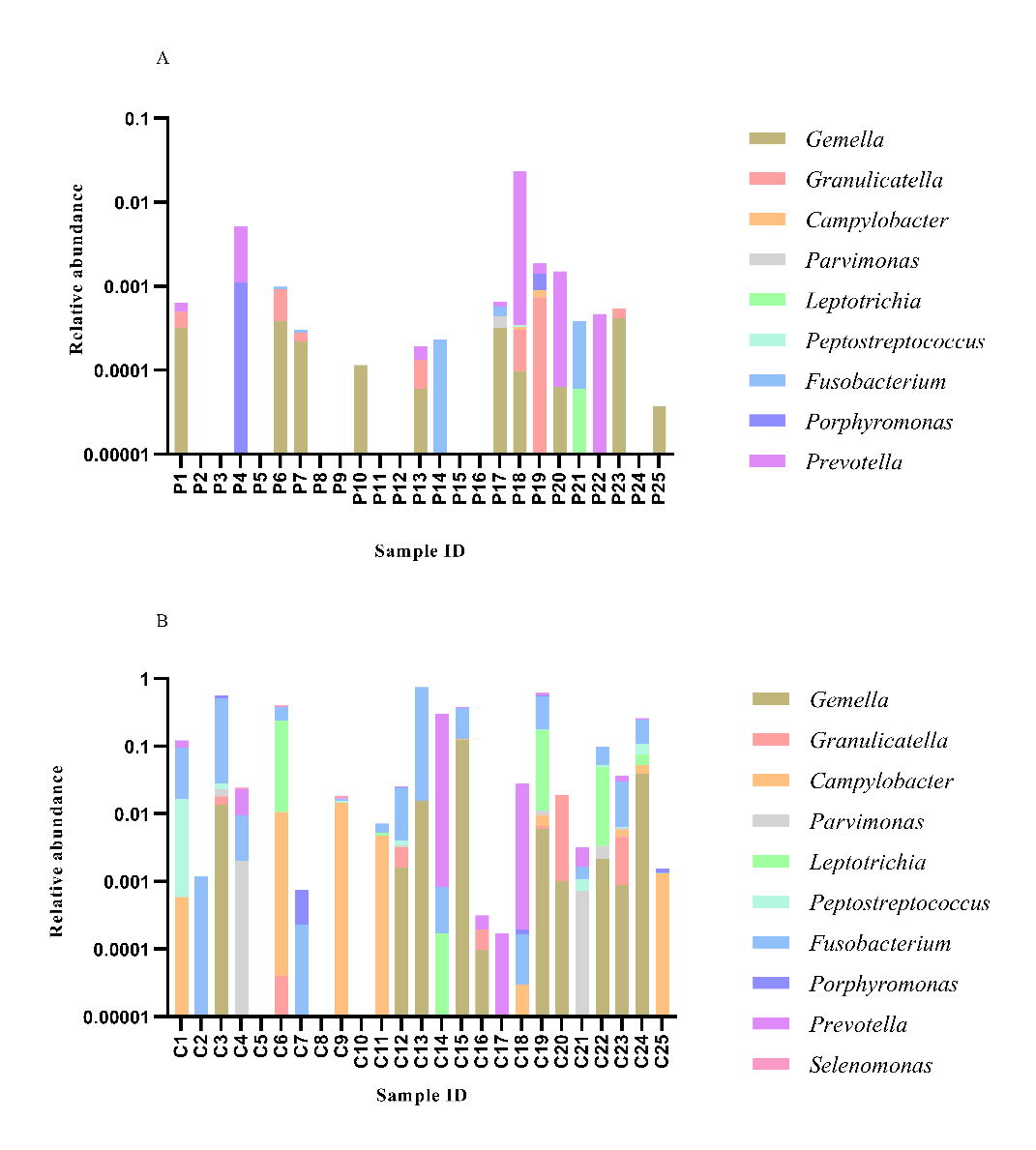


Supplementary Figure 3: Co-occurrence of biofilm associated bacteria at polyp sites in polyp patients (A) and at tumor sites in cancer patients. Relative abundance of taxa er used in the figure.
