# Supplementary material for: Microbial dynamics with CRC progression: a study of the mucosal microbiota at multiple sites in cancers, adenomatous polyps, and healthy controls
Source: Eur J Clin Microbiol Infect Dis. 2023 Jan 27;42(3):305–22. doi: 10.1007/s10096-023-04551-7 (PMC9899194; doi:10.1007/s10096-023-04551-7)
Supplement: Supplementary file 2 — Supplementary file2 (DOCX 57.1 KB) [file 10096_2023_4551_MOESM2_ESM.docx]

Supplementary Table

**Microbial dynamics with CRC progression: a study of the mucosal microbiota at multiple sites in cancers, adenomatous polyps, and healthy controls**

Thulasika Senthakumaran^1^, Aina E. F. Moen^2,3,4^, Tone M. Tannæs^2,3^, Alexander Endres^5^, Stephan A. Brackmann^5,6^, Trine B. Rounge^7^,^8^, Vahid Bemanian^9^, Hege S. Tunsjø^1^

^1^Department of Life Sciences and Health, Oslo Metropolitan University, Oslo, Norway; ^2^Section for Clinical Molecular Biology (EpiGen), Akershus University Hospital, Lørenskog, Norway; ^3^Department of Clinical Molecular Biology, Institute of Clinical Medicine, University of Oslo, Oslo, Norway; ^4^Department of Methods Development and Analytics, Norwegian Institute of Public Health, Oslo, Norway; ^5^Department of Gastroenterology, Division of Medicine, Akershus University Hospital, Lørenskog, Norway; ^6^Institute for Clinical Medicine, University of Oslo, Oslo, Norway; ^7^Centre for Bioinformatics, Department of Pharmacy, University of Oslo, Oslo, Norway; ^8^Department of Research, Cancer Registry of Norway, Oslo, Norway; ^9^Department of Pathology, Akershus University Hospital, Lørenskog, Norway

Supplementary Table 1A: Number of reads (Feature counts) per biopsy position in cancer patients

| **Sample ID** | **Feature Count** | | | |
| --- | --- | --- | --- | --- |
|  | **Ascending colon** | **Tumor** | **Healthy tissue** | **Colon sigmoideum** |
| C1 | 242162 | 200761 | 587 | 37477 |
| C2 | 137747 | 107130 | 122804 | 115694 |
| C3 | 218555 | 532329 | 9488 | 11626 |
| C4 | 92844 | 73854 | 55777 | 361588 |
| C5 | 133940 | 473 | 896 | 5530 |
| C6 | 666 | 420592 | 14688 | 165199 |
| C7 | 145488 | 60329 | 117133 | 7663 |
| C8 | 38952 | - | 95236 | 29828 |
| C9 | 267620 | 120178 | 133229 | 22454 |
| C10 | 169626 | 123384 | 83336 | 101831 |
| C11 | 51167 | 46894 | 29116 | 46227 |
| C12 | 52789 | 92127 | 33076 | 22150 |
| C13 | - | 20475 | - | - |
| C14 | 89496 | 75304 | 46172 | 101061 |
| C15 | - | 21231 | 26032 | - |
| C16 | 37282 | 50686 | 39128 | 25524 |
| C17 | 56717 | 105795 | 78919 | 80107 |
| C18 | 55686 | 66576 | 32976 | 34701 |
| C19 | 135074 | 158608 | 76492 | 80057 |
| C20 | 59980 | 82961 | 59475 | 101601 |
| C21 | - | 97054 | - | - |
| C22 | 64227 | 24342 | 100728 | 15532 |
| C23 | 42636 | 20224 | 4428 | 30179 |
| C24 | 18870 | 52512 | 9444 | 11658 |
| C25 | 63806 | 63799 | 3669 | 63845 |

Supplementary Table 1B: Number of reads (Feature counts) per biopsy position in patients with adenomatous polyps

| **Sample ID** | **Feature Count** | | | |
| --- | --- | --- | --- | --- |
|  | **Ascending colon** | **Tumor** | **Healthy tissue** | **Colon sigmoideum** |
| P1 | 141276 | 28315 | 152359 | 35 |
| P2 | 26932 | 17 | 2005 | 5800 |
| P3 | 30053 | 12565 | 35812 | 68662 |
| P4 | 81222 | 5437 | 18167 | 71315 |
| P5 | 49791 | 2825 | 172 | 363 |
| P6 | - | 112630 | 11830 | 45073 |
| P7 | 37278 | 222034 | 149534 | 214546 |
| P8 | 78223 | - | 17012 | 94803 |
| P9 | 26132 | - | - | 99878 |
| P10 | 42084 | 95497 | 198225 | 764 |
| P11 | 87829 | 27038 | 31024 | 45578 |
| P12 | 210305 | 309 | 38729 | 55242 |
| P13 | 38569 | 82852 | 35292 | - |
| P14 | 121893 | 34170 | 97411 | 44601 |
| P15 | 29439 | 8421 | 8301 | 31324 |
| P16 | 60197 |  | 5897 | 33509 |
| P17 | 24995 | 111819 | 76305 | 98626 |
| P18 | 91771 | 101801 | 108278 | 39271 |
| P19 | 132771 | 17659 | 95689 | 52386 |
| P20 | 43679 | 110786 | 84200 | 87598 |
| P21 | 41540 | 33508 | 10939 | 37891 |
| P22 | 35863 | 29787 | 29947 | - |
| P23 | 44512 | 16667 | 33394 | 10229 |
| P24 | 30261 | 17311 | 96817 | 12977 |
| P25 | 16286 | 79731 | 26036 |  |

Supplementary Table 1C: Number of reads (Feature counts) per biopsy position in control patients

| **Sample ID** | **Feature Count** | |
| --- | --- | --- |
|  | **Ascending colon** | **Colon sigmoideum** |
| K1 | 78300 | - |
| K2 | 298730 | 30110 |
| K3 | 125164 | 273083 |
| K4 | 119442 | 241474 |
| K5 | 55401 | 8389 |
| K6 | 45879 | 38235 |
| K7 | 51656 | 0 |
| K8 | 49758 | 16879 |
| K9 | 188973 | 66531 |
| K10 | 31165 | 67805 |
| K11 | 144918 | 167202 |
| K12 | 46229 | - |
| K13 | 38700 | - |
| K14 | 38820 | - |
| K15 | 40450 | 72512 |
| K16 | 53830 | 138436 |
| K17 | 84147 | 39677 |
| K18 | 38000 | 65063 |
| K19 | 81800 | 52712 |
| K20 | 53152 | 44088 |
| K21 | 26033 | 25445 |
| K22 | 24638 | 20285 |

Supplementary Table 2A: Differentially abundant ASVs between cancer and adenomatous polyp group

| Family | Genus | baseMean | log2FoldChange | padj |
| --- | --- | --- | --- | --- |
| Fusobacteriaceae | Fusobacterium | 138.4452052 | 8.78058281362985 | 3.21E-12 |
| Prevotellaceae | Prevotella | 34.18363385 | 7.3377049902771 | 0.00000159 |
| Clostridiaceae | Clostridium_perfringens | 11.48309771 | 6.25660035300987 | 0.0000842 |
| Lachnospiraceae | Family_Lachnospiraceae_5 | 21.67097865 | 4.96626603098026 | 0.003658026 |
| Peptostreptococcales-Tissierellales | Parvimonas | 10.42144441 | 4.4515479805654 | 0.00307602 |
| Gemellaceae | Gemella | 42.32536126 | 4.01754209363557 | 0.000000261 |
| Ruminococcaceae | CAG-352 | 44.52127553 | 3.94578112825772 | 0.0335865 |
| Rikenellaceae | Alistipes_1 | 68.939294 | 3.30440364003886 | 0.000000261 |
| Carnobacteriaceae | Granulicatella | 13.64439283 | 2.54303708175121 | 0.030318177 |
| Oscillospiraceae | UCG-002_1 | 53.62219057 | 2.09147444748166 | 0.010926962 |
| Lachnospiraceae | Lachnospiraceae_NK4A136_group | 80.49815509 | 1.93906225220129 | 0.044104072 |
| Christensenellaceae | Christensenellaceae_R-7_group | 27.05791744 | 1.91917358064448 | 0.039166688 |
| Rhodanobacteraceae | Rhodanobacter | 28.88647786 | 1.80116556777099 | 0.011675317 |
| Lachnospiraceae | Lachnospiraceae_UCG-010 | 103.1698925 | 1.70228479618128 | 0.036898066 |
| Lachnospiraceae | Coprococcus | 88.64465749 | 1.64570648194861 | 0.008267164 |
| Rikenellaceae | Alistipes_2 | 158.1795258 | 1.48278819129204 | 0.023365337 |
| Oscillospiraceae | UCG-002_2 | 55.98204034 | 1.36401926887434 | 0.04866015 |
| Lachnospiraceae | Blautia | 233.9653214 | 0.951767142209689 | 0.011517742 |
| Oscillospiraceae | uncultured_3 | 30.73848076 | -1.10374036327156 | 0.029754353 |
| Bacteroidaceae | Bacteroides | 111.6525022 | -2.46206907395952 | 0.003319527 |
| Tannerellaceae | Family_Tannerellaceae | 256.1958346 | -2.6199884466038 | 0.029754353 |
| Clostridiaceae | Clostridium_sensu_stricto_1 | 131.4395614 | -2.70991534414219 | 0.000499615 |
| Oscillospiraceae | Oscillibacter | 10.82389973 | -2.97071113894521 | 0.019054718 |
| Burkholderiaceae | Burkholderia-Caballeronia-Paraburkholderia | 14.26581898 | -3.91095037679994 | 0.000205192 |
| Oscillospiraceae | Colidextribacter | 36.64927028 | -5.33163109768726 | 0.00000159 |
| Lachnospiraceae | Family_Lachnospiraceae_6 | 39.57618161 | -5.48223399575318 | 0.00307602 |
| Barnesiellaceae | Barnesiella | 126.5713384 | -7.2962563795579 | 0.000338516 |
| Lachnospiraceae | Family_Lachnospiraceae_4 | 85.35142861 | -9.23663058677446 | 3.21E-12 |

Supplementary Table 2B: Differentially abundant ASVs between cancer and control group

| Family | Genus | baseMean | log2FoldChange | padj |
| --- | --- | --- | --- | --- |
| Acidaminococcaceae | Phascolarctobacterium | 139.4896083 | 27.3114219305631 | 6.45E-62 |
| Lachnospiraceae | Family_Lachnospiraceae_1 | 55.89000968 | 25.8786382449034 | 1.62E-34 |
| Desulfovibrionaceae | Desulfovibrio | 42.67018644 | 25.2237254783357 | 2.35E-20 |
| Bacteroidaceae | Bacteroides_plebeius | 14.21100822 | 24.7816103866657 | 6.87E-16 |
| Bacteroidaceae | Bacteroides_vulgatus | 94.18621424 | 24.7468316259375 | 1.61E-29 |
| Lachnospiraceae | Family_Lachnospiraceae_2 | 13.65036688 | 23.2695907563637 | 1.39E-23 |
| Fusobacteriaceae | Fusobacterium | 138.4452052 | 9.33978478645812 | 4.35E-9 |
| [Eubacterium]_copro  stanoligenes_group | [Eubacterium]_copro  stanoligenes_group | 21.8623212 | 8.10799747988757 | 8.21E-12 |
| Lachnospiraceae | Tyzzerella | 128.9255894 | 7.4910919066604 | 0.0000059 |
| Ruminococcaceae | CAG-352 | 44.52127553 | 7.34077378500124 | 0.000291713 |
| Lachnospiraceae | Frisingicoccus | 29.93554308 | 6.77854909467637 | 0.000410288 |
| uncultured | uncultured_1 | 20.52976106 | 4.86628292178721 | 0.002821003 |
| uncultured | uncultured_2 | 17.62769498 | 4.59763684681723 | 0.032468062 |
| Peptostreptococcales-  Tissierellales | Parvimonas | 10.42144441 | 4.49352959971113 | 0.027289949 |
| Gemellaceae | Gemella | 42.32536126 | 4.45150771641012 | 0.0000059 |
| Bacteroidaceae | Bacteroides_eggerthii | 107.1794062 | 4.16238667933076 | 0.042213137 |
| Oscillospiraceae | Family_Oscillospiraceae | 16.80256898 | 4.04351717865111 | 0.014084022 |
| Carnobacteriaceae | Granulicatella | 13.64439283 | 3.80600451655254 | 0.005591136 |
| Lachnospiraceae | Hungatella | 10.76755511 | 3.4620849981117 | 0.029564514 |
| Lachnospiraceae | Marvinbryantia | 6.9924223 | 3.3339962322616 | 0.045437441 |
| Lachnospiraceae | Family_Lachnospiraceae_3 | 24.19448783 | 3.06822546947065 | 0.024452496 |
| Lachnospiraceae | Lachnoclostridium | 67.73929255 | 2.09692487235144 | 0.031589409 |
| Lachnospiraceae | Dorea | 344.2302402 | 1.31202262862665 | 0.024285173 |
| Lachnospiraceae | Blautia | 660.3235152 | -0.830536536511717 | 0.04643882 |
| Lachnospiraceae | Fusicatenibacter | 300.7149389 | -1.25213243994579 | 0.048817762 |
| Enterobacteriaceae | Escherichia-Shigella | 2711.661662 | -1.96083556933837 | 0.000612287 |
| Oscillospiraceae | Colidextribacter | 36.64927028 | -5.80472984905486 | 0.0000456 |
| Lachnospiraceae | Family_Lachnospiraceae_4 | 85.35142861 | -7.21131419121846 | 0.0000501 |
| Barnesiellaceae | Barnesiella | 126.5713384 | -7.62925826000496 | 0.00426664 |

Supplementary Table 2C: Differentially abundant ASVs between polyp and control group

| Family | Genus | baseMean | log2FoldChange | padj |
| --- | --- | --- | --- | --- |
| Bacteroidaceae | Bacteroides_vulgatus | 94.18621424 | 28.1934490047552 | 4.95E-38 |
| Acidaminococcaceae | Phascolarctobacterium | 139.4896083 | 28.1683675480676 | 1.28E-65 |
| Lachnospiraceae | Family_Lachnospiraceae_1 | 55.89000968 | 26.8400238358235 | 3.76E-37 |
| Desulfovibrionaceae | Desulfovibrio | 42.67018644 | 26.5572204584177 | 1.91E-22 |
| Lachnospiraceae | Family_Lachnospiraceae_2 | 13.65036688 | 25.2846255339042 | 9.69E-28 |
| Bacteroidaceae | Bacteroides_plebeius | 14.21100822 | 24.4576486309517 | 2.31E-15 |
| Lachnospiraceae | Frisingicoccus | 29.93554308 | 7.08911839304459 | 0.000308722 |
| Burkholderiaceae | Burkholderia-Caballeronia-Paraburkholderia | 14.26581898 | 6.58778642368983 | 0.000000218 |
| Oscillospiraceae | Intestinimonas | 6.179573782 | 6.25398449000108 | 0.006251632 |
| Prevotellaceae | Massiliprevotella_massiliensis | 14.77067248 | 5.97596523180004 | 0.017254933 |
| [Eubacterium]_copros  tanoligenes_group | [Eubacterium]_copros  tanoligenes_group | 21.8623212 | 5.86782892701078 | 0.00000451 |
| Lachnospiraceae | Tyzzerella | 128.9255894 | 5.55348192181241 | 0.003782685 |
| Lachnospiraceae | Lachnospiraceae_NK4A136  _group | 6.941709124 | 5.03081718175796 | 0.020255217 |
| Bacteroidaceae | Bacteroides_eggerthii | 107.1794062 | 4.64435419593049 | 0.024764332 |
| Rikenellaceae | Alistipes_1 | 68.939294 | -2.38204475010563 | 0.017253045 |
| Prevotellaceae | Prevotella | 34.18363385 | -5.80120794088252 | 0.012826333 |
| Clostridiaceae | Clostridium_sensu_stricto_1 | 11.48309771 | -6.08832645114385 | 0.006399578 |

Supplementary Table 3A: Significantly differentially abundant genera between cancer and polyp group

| **Family** | **Genus** | **baseMean** | **log2FoldChange** | **padj** |
| --- | --- | --- | --- | --- |
| Fusobacteriaceae | Fusobacterium | 308.4975277 | 8.681840902 | 1.45693E-29 |
| Peptostreptococcaceae | Peptostreptococcus | 28.80220484 | 7.246412151 | 0.00000000330735 |
| Gemellaceae | Gemella | 36.00014476 | 3.796022185 | 0.000000717997 |
| Enterobacteriaceae | Family_Enterobacteriaceae | 102.9853166 | -7.656496988 | 0.00000329 |
| Leptotrichiaceae | Leptotrichia | 25.16556504 | 7.811370904 | 0.00000491813 |
| Comamonadaceae | Family_Comamonadaceae | 8.277131875 | -3.612239449 | 0.0000077474 |
| Prevotellaceae | Prevotella | 848.4641812 | 3.508009771 | 0.0000660925 |
| Burkholderiaceae | Burkholderia-Caballeronia-Paraburkholderia | 13.98948854 | -3.786921986 | 0.000192861 |
| Peptostreptococcales-Tissierellales | Parvimonas | 8.858017935 | 4.334750287 | 0.000374293 |
| Selenomonadaceae | Selenomonas | 9.001918397 | 7.686784761 | 0.000435335 |
| Campylobacteraceae | Campylobacter | 6.580867057 | 4.378977046 | 0.001009305 |
| Caulobacteraceae | Caulobacter | 1.612360272 | -3.789631069 | 0.002043277 |
| Porphyromonadaceae | Porphyromonas | 17.81008258 | 4.489564386 | 0.00249768 |
| Butyricicoccaceae | UCG-008 | 4.22216014 | -3.557344757 | 0.00313698 |
| Oscillospiraceae | NK4A214_group | 27.08205487 | 1.66207587 | 0.00313698 |
| Micropepsaceae | uncultured | 2.00755098 | -4.394266989 | 0.003346762 |
| Microbacteriaceae | Leifsonia | 3.422664029 | -3.272757873 | 0.00369364 |
| Veillonellaceae | Family_Veillonellaceae | 3.095771387 | 5.143119891 | 0.015455631 |
| Oscillospiraceae | Flavonifractor | 212.2903973 | -1.047334856 | 0.015455631 |
| Oscillospiraceae | Colidextribacter | 193.7846814 | -0.767490233 | 0.015455631 |
| Ruminococcaceae | Family_Ruminococcaceae | 17.08624538 | 1.302314595 | 0.018790533 |
| Tannerellaceae | Family_Tannerellaceae | 247.0594904 | -2.581950659 | 0.020857717 |
| Oscillospiraceae | UCG-002 | 201.5394138 | 0.942311836 | 0.025414678 |
| Lachnospiraceae | Lachnospiraceae_UCG-010 | 128.2355151 | 1.338809931 | 0.033957194 |
| Lachnospiraceae | Coprococcus | 781.1667141 | 0.859936096 | 0.036898249 |
| Anaerovoracaceae | Family_XIII_AD3011_group | 12.62609629 | 1.030200546 | 0.036976275 |
| Tannerellaceae | Parabacteroides | 909.372743 | -0.934921985 | 0.043093484 |

Supplementary Table 3B: Significantly differentially abundant genera between cancer and control group

| **Family** | **Genus** | **baseMean** | **log2FoldChange** | **padj** |
| --- | --- | --- | --- | --- |
| Lachnospiraceae | Butyrivibrio | 10.07997673 | 24.09522691 | 5.67139E-34 |
| Fusobacteriaceae | Fusobacterium | 308.4975277 | 7.449753805 | 9.05565E-14 |
| Clostridiaceae | Clostridium_sensu  _stricto_1 | 350.4288738 | -3.981467982 | 1.69118E-09 |
| Peptostreptococcaceae | Peptostreptococcus | 28.80220484 | 8.104493756 | 2.74866E-07 |
| Leptotrichiaceae | Leptotrichia | 25.16556504 | 9.685144197 | 0.0000085532 |
| Gemellaceae | Gemella | 36.00014476 | 4.217154202 | 0.0000159628 |
| Oscillospiraceae | Colidextribacter | 193.7846814 | -1.494470469 | 0.0000239729 |
| Porphyromonadaceae | Porphyromonas | 17.81008258 | 6.878011366 | 0.00014676 |
| Enterobacteriaceae | Escherichia-Shigella | 2933.307128 | -2.095571716 | 0.000183872 |
| Campylobacteraceae | Campylobacter | 6.580867057 | 5.540890901 | 0.001143508 |
| Selenomonadaceae | Selenomonas | 9.001918397 | 8.215779919 | 0.005291873 |
| Carnobacteriaceae | Granulicatella | 13.97363413 | 3.230941288 | 0.013224679 |
| Oscillospiraceae | Flavonifractor | 212.2903973 | -1.351065429 | 0.013224679 |
| Oscillospiraceae | NK4A214_group | 27.08205487 | 1.827319027 | 0.013224679 |
| Prevotellaceae | Prevotella | 848.4641812 | 3.011347673 | 0.013224679 |
| Lachnospiraceae | Fusicatenibacter | 324.3644405 | -1.465734591 | 0.013224679 |
| Lachnospiraceae | Tyzzerella | 196.678357 | 3.551574873 | 0.013224679 |
| [Eubacterium]_copro  stanoligenes_group | [Eubacterium]_copro  stanoligenes_group | 63.1332441 | 1.520443918 | 0.01693078 |
| Pseudomonadaceae | Pseudomonas | 6.262193787 | -3.835587866 | 0.017373646 |
| Peptostreptococcales-Tissierellales | Parvimonas | 8.858017935 | 3.923281545 | 0.017373646 |
| Lachnospiraceae | Lachnospiraceae_  ND3007_group | 73.72212037 | -1.424643992 | 0.018355045 |
| Veillonellaceae | Dialister | 21.30277491 | 2.572306934 | 0.021184123 |
| Lachnospiraceae | Hungatella | 26.90956771 | 2.437449406 | 0.02151477 |
| Izemoplasmatales | Izemoplasmatales | 4.100078058 | 3.991273262 | 0.024097414 |
| Lachnospiraceae | Lachnospiraceae_  UCG-010 | 128.2355151 | 1.703528759 | 0.026010778 |
| Enterobacteriaceae | Family_Enterobacteriaceae | 102.9853166 | -4.815477046 | 0.045122659 |
| Lachnospiraceae | uncultured | 262.7583475999 | -0.981553249 | 0.047367459 |

Supplementary Table 3C: Significantly differentially abundant genera between polyp and control group

| **Family** | **Genus** | **baseMean** | **log2FoldChange** | **padj** |
| --- | --- | --- | --- | --- |
| Lachnospiraceae | Butyrivibrio | 10.07997673 | 23.21387069 | 3.56451E-31 |
| Burkholderiaceae | Burkholderia-Caballeronia-Paraburkholderia | 13.98948854 | 6.697605825 | 2.17729E-07 |
| Comamonadaceae | Family_Comamonadaceae | 8.277131875 | 4.991336212 | 0.00000433117 |
| Clostridiaceae | Clostridium_sensu_stricto_1 | 350.4288738 | -2.943220354 | 0.0000592134 |
| Microbacteriaceae | Leifsonia | 3.422664029 | 5.308440815 | 0.000335906 |
| Ruminococcaceae | Family_Ruminococcaceae | 17.08624538 | -1.884378123 | 0.016742162 |
| Caulobacteraceae | Caulobacter | 1.612360272 | 4.280841081 | 0.02540073 |
| Isosphaeraceae | uncultured | 2.093990913 | 4.404099114 | 0.033483927 |
| Ruminococcaceae | Fournierella | 16.1600742 | 3.517844259 | 0.033483927 |
| Enterobacteriaceae | Escherichia-Shigella | 2933.307128 | -1.498056433 | 0.040633594 |

Supplementary Tabell 4: Taxonomic classification of *Fusobacterium* spp. using *zink protease* and 16S rRNA gene v3 amplicon reads from MinION

| Prøve ID | Taxon ID | Target | Number of *Fusobacterium* Reads^a)^ | Relative abundance of *Fusobacterim*^b)^ |
| --- | --- | --- | --- | --- |
| C19 | [*Fusobacterium nucleatum* ssp. *animalis*](https://www.ncbi.nlm.nih.gov/Taxonomy/Browser/wwwtax.cgi?mode=Info&id=469607)  [*Fusobacterium nucleatum* ssp. *vincentii*](https://www.ncbi.nlm.nih.gov/Taxonomy/Browser/wwwtax.cgi?mode=Info&id=155615) | *zinc protease* | 132 057  16 457 | 83,5 %  10.4 % |
| C8 | [*Fusobacterium nucleatum* ssp. *animalis*](https://www.ncbi.nlm.nih.gov/Taxonomy/Browser/wwwtax.cgi?mode=Info&id=76859) | *zinc protease* | 86 275 | 96,3 % |
| C24 | [*Fusobacterium nucleatum* ssp. *nucleatum*](https://www.ncbi.nlm.nih.gov/Taxonomy/Browser/wwwtax.cgi?mode=Info&id=76856) | *zinc protease* | 68142 | 99,2 % |
| C3 | [*Fusobacterium nucleatum* ssp. *animalis*](https://www.ncbi.nlm.nih.gov/Taxonomy/Browser/wwwtax.cgi?mode=Info&id=76859) | *zinc protease* | 44 458 | 91,0 % |
| C13 | [*Fusobacterium nucleatum* ssp. *animalis*](https://www.ncbi.nlm.nih.gov/Taxonomy/Browser/wwwtax.cgi?mode=Info&id=76859) | *zinc protease* | 37 883 | 96,5 % |
| C4 | [*Fusobacterium nucleatum* ssp. *animalis*](https://www.ncbi.nlm.nih.gov/Taxonomy/Browser/wwwtax.cgi?mode=Info&id=76859)  [*Fusobacterium nucleatum* ssp. *vincentii*](https://www.ncbi.nlm.nih.gov/Taxonomy/Browser/wwwtax.cgi?mode=Info&id=155615) | *zinc protease* | 8724  1044 | 83,9 %  10,0 % |
| C22 | [*Fusobacterium nucleatum* ssp. *animalis*](https://www.ncbi.nlm.nih.gov/Taxonomy/Browser/wwwtax.cgi?mode=Info&id=76859) | *zinc protease* | 3838 | 85 % |
| C6 | [*Fusobacterium nucleatum* ssp*. animalis*](https://www.ncbi.nlm.nih.gov/Taxonomy/Browser/wwwtax.cgi?mode=Info&id=76859)  [*Fusobacterium nucleatum* ssp. *vincentii*](https://www.ncbi.nlm.nih.gov/Taxonomy/Browser/wwwtax.cgi?mode=Info&id=155615)  [*Fusobacterium nucleatum*](https://www.ncbi.nlm.nih.gov/Taxonomy/Browser/wwwtax.cgi?mode=Info&id=851) | *zinc protease* | 1789  640  2021 | 39,6 %  14,2 %  44,8 % |
| C15 | [*Fusobacterium nucleatum* ssp. *animalis*](https://www.ncbi.nlm.nih.gov/Taxonomy/Browser/wwwtax.cgi?mode=Info&id=76859) | *zinc protease* | 1842 | 87,4 % |
| C14 | [*Fusobacterium nucleatum* ssp. *animalis*](https://www.ncbi.nlm.nih.gov/Taxonomy/Browser/wwwtax.cgi?mode=Info&id=76859) | *zinc protease* | 805 | 96 % |
| C12 | [*Fusobacterium pseudoperiodonticum*](https://www.ncbi.nlm.nih.gov/Taxonomy/Browser/wwwtax.cgi?mode=Info&id=2663009) | *zinc protease* | 856 | 90,1 % |
| C9 | [*Fusobacterium nucleatum* ssp*. animalis*](https://www.ncbi.nlm.nih.gov/Taxonomy/Browser/wwwtax.cgi?mode=Info&id=76859) | *zinc protease* | 208 | 89,6 % |
| C2^c)^ | *Fusobacterium necrophorum*  *Fusobacterium gonidiaformans*  *Fusobacterium nucleatum* | 16S rRNA V3 | 5646  86  40 | 89%  1,3%  0,6% |
| C2 | [*Fusobacterium nucleatum* ssp. *animalis*](https://www.ncbi.nlm.nih.gov/Taxonomy/Browser/wwwtax.cgi?mode=Info&id=76859) | *zinc protease* | 338 | 81 % |
| C23 | [*Fusobacterium nucleatum* ssp. *animalis*](https://www.ncbi.nlm.nih.gov/Taxonomy/Browser/wwwtax.cgi?mode=Info&id=76859) | *zinc protease* | 1 |  |
| C23^c)^ | *Fusobacterium gonidiaformans* | 16S rRNA V3 | 996 | 87,7% |
| C1 | [*Fusobacterium nucleatum* ssp.](https://www.ncbi.nlm.nih.gov/Taxonomy/Browser/wwwtax.cgi?mode=Info&id=76859) *vincentii*  *Fusobacterium nucleatum* | *zinc protease* | 10  3 | 62,5 %  18% |
| C1^c)^ | *Fusobacterium nucleatum* | 16S rRNA V3 | 446 | 70,4% |
| C5 | [*Fusobacterium nucleatum* ssp.](https://www.ncbi.nlm.nih.gov/Taxonomy/Browser/wwwtax.cgi?mode=Info&id=76859) *polymorphum*  *Fusobacterium nucleatum* | *zinc protease* | 9  9 | 47%  47% |
| C5^c)^ | *Fusobacterium sp.*  *Fusobacterium canelifelinum*  *Fusobacterium nucleatum*  (Reported as *Fusobacterium sp*) | 16S rRNA V3 | 45  29  42 | 38%  25%  36% |
| C11 | [*Fusobacterium nucleatum* ssp. *animalis*](https://www.ncbi.nlm.nih.gov/Taxonomy/Browser/wwwtax.cgi?mode=Info&id=76859)  [*Fusobacterium nucleatum* ssp.](https://www.ncbi.nlm.nih.gov/Taxonomy/Browser/wwwtax.cgi?mode=Info&id=76859) *vincentii* | *zinc protease* | 13  17 | 33 %  43% |
| C11^c)^ | *Fusobacterium nucleatum*  [*Fusobacterium nucleatum* ssp. *animalis*](https://www.ncbi.nlm.nih.gov/Taxonomy/Browser/wwwtax.cgi?mode=Info&id=76859) (Reported as *Fusobacterium nucleatum*) | 16S rRNA V3 | 91  26 | 56.8%  31.1 % |
| C21^c)^ | *Fusobacterium canelifelinum*  [*Fusobacterium nucleatum* ssp*.*](https://www.ncbi.nlm.nih.gov/Taxonomy/Browser/wwwtax.cgi?mode=Info&id=76859) *Polymorphum*  (Uncertain results) | 16S rRNA V3 | 3  1 |  |
| C21 | [*Fusobacterium nucleatum* ssp*. animalis*](https://www.ncbi.nlm.nih.gov/Taxonomy/Browser/wwwtax.cgi?mode=Info&id=76859)  [*Fusobacterium nucleatum* ssp*.*](https://www.ncbi.nlm.nih.gov/Taxonomy/Browser/wwwtax.cgi?mode=Info&id=76859) *polymorphum* | *zinc protease* | 11  5 | 58 %  26 % |
| [*F. nucleatum* ssp. *animalis*](https://www.ncbi.nlm.nih.gov/Taxonomy/Browser/wwwtax.cgi?mode=Info&id=76859) *CCUG 32879* | [*Fusobacterium nucleatum* ssp. *animalis*](https://www.ncbi.nlm.nih.gov/Taxonomy/Browser/wwwtax.cgi?mode=Info&id=76859)  [*Fusobacterium nucleatum* ssp. *vincentii*](https://www.ncbi.nlm.nih.gov/Taxonomy/Browser/wwwtax.cgi?mode=Info&id=155615)  [*Fusobacterium nucleatum* ssp. *nucleatum*](https://www.ncbi.nlm.nih.gov/Taxonomy/Browser/wwwtax.cgi?mode=Info&id=76856) | *zinc protease* | 61525 | 92,3 %  4%  0,5% |
| *F. nucleatum* ssp. *polymorphum CCUG 9126T* | [*Fusobacterium nucleatum ssp.*](https://www.ncbi.nlm.nih.gov/Taxonomy/Browser/wwwtax.cgi?mode=Info&id=76859) *polymorphum*  *Fusobacterium nucleatum* | *zinc protease* | 13142  4524 | 73%  25% |
| *F. nucleatum* ssp. *vincentii CCUG 37843T* | [*Fusobacterium nucleatum* ssp.](https://www.ncbi.nlm.nih.gov/Taxonomy/Browser/wwwtax.cgi?mode=Info&id=76859) *vincentii*  *Fusobacterium nucleatum* | *zinc protease* | 11625  68636 | 14,4%  84,9% |
| *F. nucleatum* ssp. *nucleatum CCUG 33059* | *Fusobacterium nucleatum* ssp. *nucleatum* | *zinc protease* | 79541 | 98% |
| [*F. nucleatum* ssp. *animalis*](https://www.ncbi.nlm.nih.gov/Taxonomy/Browser/wwwtax.cgi?mode=Info&id=76859) *CCUG 32879*^c)^ | [*Fusobacterium nucleatum*](https://www.ncbi.nlm.nih.gov/Taxonomy/Browser/wwwtax.cgi?mode=Info&id=851)  [*Fusobacterium canifelinum*](https://www.ncbi.nlm.nih.gov/Taxonomy/Browser/wwwtax.cgi?mode=Info&id=285729)  [*Fusobacterium nucleatum* ssp. *animalis*](https://www.ncbi.nlm.nih.gov/Taxonomy/Browser/wwwtax.cgi?mode=Info&id=76859)  [*Fusobacterium nucleatum* ssp. *vincentii*](https://www.ncbi.nlm.nih.gov/Taxonomy/Browser/wwwtax.cgi?mode=Info&id=155615) | 16S rRNA V3 | 1867  413  328  299 | 53,29  11,7%  9,3%  8,5% |

a) Number of *zink protease* (Kim et al) sequence reads from MinION, classified as *Fusobacterium* spp. using WIMP. b) Abundance of the *Fusobacterium* sp./ssp. relative to the total number of reads classified as *Fusobacterium* spp. c) Identification obtained with *Fusobacterium* specific 16S rRNA V3 amplicons (Walter et al). The *zink protease* target (specific for *F. nucleatum*/*F. periodonticum*/*F. pseudoperiodonticum)* did not reveal any *Fusobacterium* sequences from these samples.
